# Gut-associated IgA^+^ immune cells regulate obesity-related insulin resistance

**DOI:** 10.1038/s41467-019-11370-y

**Published:** 2019-08-13

**Authors:** Helen Luck, Saad Khan, Justin H. Kim, Julia K. Copeland, Xavier S. Revelo, Sue Tsai, Mainak Chakraborty, Kathleen Cheng, Yi Tao Chan, Mark K. Nøhr, Xavier Clemente-Casares, Marie-Christine Perry, Magar Ghazarian, Helena Lei, Yi-Hsuan Lin, Bryan Coburn, Allan Okrainec, Timothy Jackson, Susan Poutanen, Herbert Gaisano, Johane P. Allard, David S. Guttman, Margaret E. Conner, Shawn Winer, Daniel A. Winer

**Affiliations:** 1 Division of Cellular and Molecular Biology, Diabetes Research Group, Toronto General Research Institute (TGRI), University Health Network, Toronto, ON M5G 2C4 Canada; 2 Department of Immunology, University of Toronto, Toronto, ON M5S 1A8 Canada; 3 Centre for the Analysis of Genome Evolution and Function, University of Toronto, Toronto, ON M5S 3B2 Canada; 4 Department of Integrative Biology and Physiology, University of Minnesota, Minneapolis, MN 55455 USA; 5 Center for Immunology, University of Minnesota, Minneapolis, MN 55455 USA; 6 Department of Medical Microbiology and Immunology, University of Alberta, Edmonton, AB T6G 2R3 Canada; 7 Center for Traditional Chinese Medicine, Chang Gung Memorial Hospital, School of Traditional Chinese Medicine and Graduate Institute of Clinical Medical Sciences, College of Medicine, Chang Gung University, Taoyuan, 333 Taiwan; 8 Department of Medicine, Division of Infectious Diseases, University Health Network, Toronto, ON M5G 2N2 Canada; 9 Department of Laboratory Medicine and Pathobiology, University of Toronto, Toronto, ON M5S 1A8 Canada; 10 Toronto Western Hospital, University Health Network, Toronto, ON M5T 2S8 Canada; 11 Department of Surgery, University of Toronto, Toronto, ON M5T 1P5 Canada; 12 Department of Medicine, University of Toronto, Toronto, ON M5S 1A8 Canada; 13 Department of Microbiology, University Health Network/Sinai Health System, Toronto, ON M5G 2M9 Canada; 14 Toronto General Hospital, University Health Network, Toronto, ON M5G 2C4 Canada; 15 Department of Virology and Microbiology, Baylor College of Medicine, Houston, TX 77030 USA; 16 Department of Laboratory Medicine, St. Michael’s Hospital, Toronto, ON M5B 1W8 Canada; 17 Department of Pathology, University Health Network, 200 Elizabeth Street, Toronto, ON M5G 2C4 Canada; 18 Buck Institute for Research on Aging, 8001 Redwood Boulevard, Novato, CA 94945 USA; 19 Present Address: 10-352 Toronto Medical Discovery Tower, 101 College Street, Toronto, ON M5G 1L7 Canada

**Keywords:** Mucosal immunology, Diabetes, B cells, Obesity

## Abstract

The intestinal immune system is emerging as an important contributor to obesity-related insulin resistance, but the role of intestinal B cells in this context is unclear. Here, we show that high fat diet (HFD) feeding alters intestinal IgA^+^ immune cells and that IgA is a critical immune regulator of glucose homeostasis. Obese mice have fewer IgA^+^ immune cells and less secretory IgA and IgA-promoting immune mediators. HFD-fed IgA-deficient mice have dysfunctional glucose metabolism, a phenotype that can be recapitulated by adoptive transfer of intestinal-associated pan-B cells. Mechanistically, IgA is a crucial link that controls intestinal and adipose tissue inflammation, intestinal permeability, microbial encroachment and the composition of the intestinal microbiome during HFD. Current glucose-lowering therapies, including metformin, affect intestinal-related IgA^+^ B cell populations in mice, while bariatric surgery regimen alters the level of fecal secretory IgA in humans. These findings identify intestinal IgA^+^ immune cells as mucosal mediators of whole-body glucose regulation in diet-induced metabolic disease.

## Abstract (web summary)

The effect of diet-induced obesity on intestinal B cell populations is not well understood despite emerging evidence of a critical role for the intestinal immune system in contributing to insulin resistance. Here, the authors show important functions of IgA in regulating metabolic disease and for intestinal immunity in modulating systemic glucose metabolism.

## Introduction

Obesity is a global concern that is associated with many chronic complications such as type 2 diabetes, insulin resistance (IR), cardiovascular diseases, and cancer. Growing evidence has implicated the digestive system, including its microbiota, gut-derived incretin hormones, and gut-associated lymphoid tissue in obesity and IR^1^. During high fat diet (HFD) feeding and obesity, a significant shift occurs in the microbial populations within the gut, known as dysbiosis, which interacts with the intestinal immune system. Similar to other metabolic organs, including visceral adipose tissue (VAT) and liver^2–5^, altered immune homeostasis has also been observed in the small and large intestines during obesity^6–10^.

During diet-induced obesity (DIO), many subsets of adaptive immune cells within the gut adopt a pro-inflammatory phenotype, primarily demonstrated by T cells producing pro-inflammatory cytokines, such as interferon-γ (IFNγ)^7,8^. This shift is accompanied by a reduction in proportions of regulatory T cells (Tregs) and other immune cells, including interleukin-22 (IL-22) producing innate lymphoid cells (ILCs) and T-helper type 17 (Th17) T cells, which help to maintain intestinal homeostasis^7,8,11,12^. In addition to microbial dysbiosis, HFD feeding is associated with aberrant mucosal barrier function, subsequent bacterial product leakage, and metabolic endotoxemia^6,13^. Despite these findings, changes in additional gut immune populations, including B and plasma cells, during DIO and mechanisms behind intestinal immune regulation of glucose homeostasis remain unclear.

A link between the gut microbiota and the intestinal immune system is the immune-derived molecule immunoglobulin A (IgA). IgA is a B cell antibody primarily produced in dimeric form by plasma cells residing in the gut lamina propria (LP). The production of IgA is influenced by innate immune cells including dendritic cells (DCs) and pathogen sensing pathways, such as Toll-like receptor 2^14–17^. IgA can also be produced by B1 cells, and these cells may modulate IR^18^. In conjunction with antimicrobial peptides, mucus and host defense molecules, IgA is essential for maintaining gut homeostasis through protection of the mucosal surface from pathogens while tolerating commensal bacteria using mechanisms such as immune exclusion^19^. IgA directly binds to commensal bacteria together with the mucous layer to prevent bacteria from breaching the intestinal epithelial barrier, and also assists in the expulsion of bacterial components that have intruded the epithelial barrier^20^.

Given the importance of IgA on intestinal–gut microbe immunoregulation, which is directly influenced by dietary changes, we hypothesized that IgA may be a key player in the pathogenesis of obesity and IR. Here, we demonstrate that IgA levels are reduced during obesity and the loss of IgA in mice worsens IR and increases intestinal permeability, microbiota encroachment, and downstream inflammation in metabolic tissues, including inside the VAT. IgA deficiency alters the obese gut microbiota and its metabolic phenotype can be recapitulated into microbiota-depleted mice upon fecal matter transplantation. In addition, we show that commonly used therapies for diabetes such as metformin and bariatric surgery can alter cellular and stool IgA levels, respectively. Our findings suggest a critical function for IgA in regulating metabolic disease and support the emerging role for intestinal immunity as an important modulator of systemic glucose metabolism.

## Results

### Intestinal IgA^+^ immune cells are altered in HFD-fed mice

**Fig. 1:**
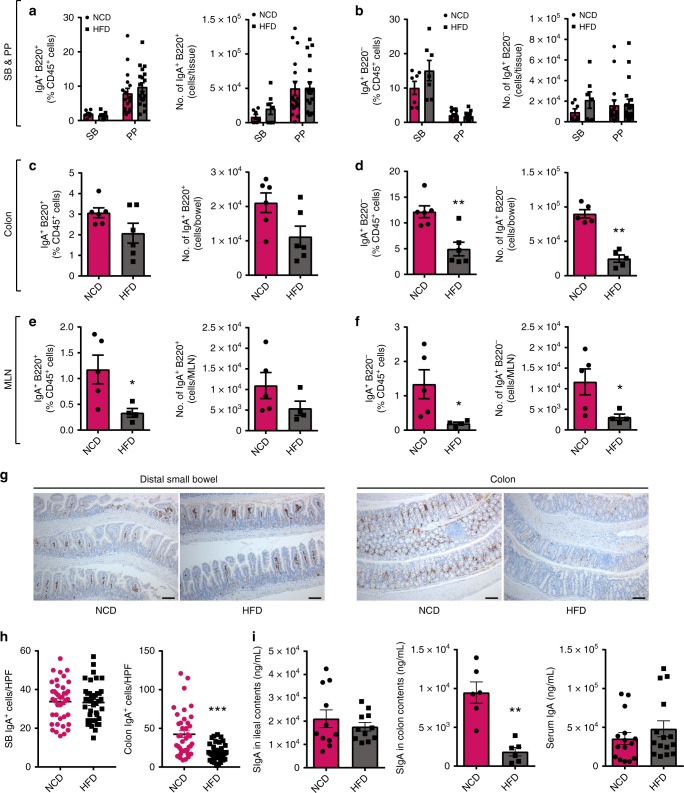
The number of immunoglobulin A (IgA)-producing cells is reduced within the intestinal immune system of high fat diet (HFD)-fed mice. Frequency and absolute number of IgA-producing B cells (IgA^+^ B220^+^) and plasma cells (IgA^+^ B220^−^) within the **a**, **b** distal small intestine LP (SB) (*n* = 7/group, 2 experiments), Peyer’s patches (PP) (*n* = 17–19/group, 5 experiments), **c**, **d** colon LP (*n* = 6/group, 2 experiments), and **e**, **f** colon draining mesenteric lymph nodes (MLNs) (*n* = 4–5/group) in HFD-fed C57BL/6J mice after 14 weeks compared to normal control diet (NCD)-fed controls. **g** Representative staining of IgA^+^ cells within the distal SB (left) and colon (right) in HFD- and NCD-fed mice after 14 weeks of diet (scale bar = 100 µm). **h** Enumeration of IgA+ cells per ×400 high power field (HPF) in the distal SB (left) and colon (right) in NCD and HFD-fed mice (*n* = 4/group, at least 10 HPF counted per mouse). **i** Concentration of secretory IgA (SIgA) within ileal contents (left) (*n* = 11/group) and colonic stool (middle) (*n* = 6/group), and IgA antibody titers in the serum (right) (*n* = 15/group) of NCD and HFD wild-type (WT) mice. Data are means ± SEM. * denotes *p* < 0.05, ** denotes *p* < 0.01, and *** denotes *p* < 0.001

To identify changes in intestinal B cell populations during obesity, C57BL/6 mice were fed a HFD for 12–14 weeks followed by assessment of the intestinal LP by flow cytometry. The percentage and absolute cell numbers of B cell populations, including IgM and IgG expressing B2 populations and B1 cells, were unchanged in the small and large intestine LP in HFD-fed mice, compared to normal control diet (NCD)-fed controls (Supplementary Fig. 1a–d). No differences were observed in the percentage or number of IgA-producing B (IgA^+^ B220^+^) (Fig. 1a) and plasma (IgA^+^ B220^−^) cells (Fig. 1b) within the distal small intestine LP and Peyer’s patches. Representative gating strategies for IgA^+^ B220^+^ and IgA^+^ B220^−^ cells and other immune cell populations analyzed in this study are shown in Supplementary Fig. 10. In contrast, the frequency and number of IgA-producing plasma cells, but not IgA^+^ B cells, in the colonic LP were reduced in HFD-fed mice (Fig. 1c, d). A reduction in the percentage of IgA^+^ B cells (Fig. 1e) and percentage and number of IgA-producing plasma cells (Fig. 1f) was identified in the mesenteric lymph nodes (MLNs) during HFD feeding. Consistent with the flow cytometry, immunohistochemistry confirmed that IgA^+^-stained cells were reduced in colonic tissue of HFD-fed mice compared to NCD controls, while IgA^+^ cells were unchanged in the distal small bowel (Fig. 1g, h). In addition, secretory IgA (SIgA) concentrations within the luminal contents of the ileum were unchanged (Fig. 1i, left), but were significantly decreased in the colonic luminal contents of HFD-fed mice (Fig. 1i, middle). No differences were detected in the serum concentration of IgA between NCD- and HFD-fed mice (Fig. 1i, right). Overall, the results indicate that HFD feeding affects IgA-producing cells within the gut-associated immune system, as well as SIgA in the colon.

### HFD feeding alters immune regulators of IgA production

**Fig. 2:**
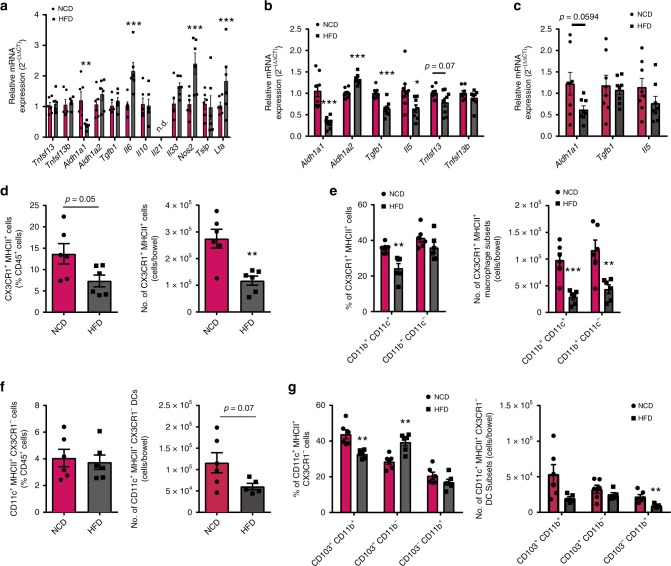
High fat diet (HFD) feeding impedes secreted factors and immune cells promoting intestinal immunoglobulin A (IgA). Relative messenger RNA (mRNA) expression of genes promoting IgA in colon **a** whole tissue (*n* = 6/group), **b** lamina propria (LP) (*n* = 8/group, 2 experiments), and **c** epithelial cell fraction (*n* = 8/group, 2 experiments) in HFD-fed C57BL/6J mice after 14 weeks compared to normal control diet (NCD) controls. Frequency (left) and absolute number (right) of **d** CX3CR1^+^ MHCII^+^ macrophages, **e** corresponding macrophage subsets, **f** CD11c^+^ MHCII^+^ CX3CR1^−^ dendritic cells, and **g** corresponding dendritic cell subsets in the colonic LP of C57BL/6J mice fed a HFD/NCD for 14 weeks (*n* = 6/group, 2 experiments). Data are means ± SEM. * denotes *p* < 0.05, ** denotes *p* < 0.01, and *** denotes *p* < 0.001

We next sought to delineate mechanisms behind the site-specific reduction of IgA^+^ cells in the colon and colonic-associated MLN during HFD feeding. Using a custom gene expression assay containing secreted factors that mediate plasma cell differentiation and/or IgA class-switch recombination (CSR), we first identified that whole colon tissue from HFD-fed mice showed a large decrease in the expression of *Aldh1a1*, a gene encoding retinaldehyde dehydrogenase 1 (ALDH1A1) (Fig. 2a). ALDH1A1 is an important enzyme in the synthesis of retinoic acid (RA), which affects IgA production^21^. We observed increases in gene expression of *Il6*, *Nos2*, and *Lta*, which indirectly stimulate IgA class switching and plasma cell differentiation, but are also elevated during pro-inflammatory conditions and activated by the nuclear factor-κB pathway^22,23^. Similarly, gene expression of *Nos2*, *Lta*, and *Tslp* was increased in the small intestine tissue (Supplementary Fig. 2a).

To gain further insight into the cellular source of altered IgA-related gene expression within the intestines, we performed gene expression analysis on cell suspensions from LP and epithelial fractions. Within the HFD-fed colonic LP, there was a significant decrease in the expression of IgA promoting factors: *Aldh1a1*, *Il5*, *Tgfb1*, and a trending reduction in *Tnfsf13* (APRIL) (Fig. 2b). Transforming growth factor-β1 (TGF-β1) is an essential IgA CSR factor, which is necessary for both T-dependent (TD) and T-independent (TI) IgA class switching^24–26^. IL-5 can enhance IgA-promoting functions of TGF-β1 as well as RA, in addition to stimulating the maturation of B cells into differentiated plasma cells^27–29^. APRIL is also involved in enhancing IgA CSR and mice deficient in APRIL possess impaired IgA responses^30^. Although a small increase in the expression of *Aldh1a2* was observed, this change may reflect homeostatic compensation for the marked ~70% decrease in the expression of its family member, *Aldh1a1*. In the HFD-fed colonic epithelial cell fraction, we found a trending reduction in the expression of *Aldh1a1* with no alterations in the expression of *Tgfb1* and *Il5* (Fig. 2c). No changes in gene expression were observed in the small intestine (LP and epithelium), with the exception of a similar minor increase in *Aldh1a2*, and *Tnfs13b* (BAFF) in the small intestinal LP (Supplementary Fig. 2b, c). These data support our previous findings regarding intestinal site-specific loss in IgA populations, as reductions in IgA promoting factors were observed exclusively in the colon upon HFD feeding.

We next characterized HFD-induced changes to the innate myeloid immune compartment within the LP, as they are a source of TGF-β1, IL-5, APRIL, and RA, linked to IgA production^31^. HFD-fed mice displayed a decrease in colonic CX3CR1^+^ macrophages in the LP (Fig. 2d). Additionally, in the colon, HFD feeding induced a decrease in the frequency and number of the IgA inducing CD11b^+^ CD11c^+^ macrophage subset, as well as a decrease in the number of CD11b^+^ CD11c^−^ macrophages, which have been linked to the regulation of Treg responses, which are also dampened during DIO (Fig. 2e)^8,32,33^. Alternatively, in the small intestine, while the frequency and numbers of CX3CR1^+^ macrophages and its CD11b^+^ CD11c^−^ subset were decreased, no changes were seen in the CD11b^+^ CD11c^+^ macrophage compartment (Supplementary Fig. 2d, e). HFD feeding did not alter total CD11c^+^ MHCII^+^ CX3CR1^−^ DCs in the colon (Fig. 2f), but decreased the proportions of CD103^+^ CD11b^+^ DC subset known to promote IgA responses^34^ while increasing the proportions of CD103^+^ CD11b^−^ DCs which was previously shown to enhance intestinal CD8^+^ and Th1 responses^35,36^ (Fig. 2g). In contrast to the colon, the small intestine of HFD mice had increased proportions of total CD11c^+^ MHCII^+^ CX3CR1^−^ DCs, yet displayed no differences in the frequencies and proportions of their various subsets (Supplementary Fig. 2f, g). In the PP, HFD feeding led to a trending loss in the frequency of DCs, and an increase in the number of total CX3CR1^+^ macrophages, but no differences were observed in the gene expression of IgA-promoting factors, or macrophage and DC subsets (Supplementary Fig. 2h–l). In the colon-associated MLN, we observed a decreased expression of *Aldh1a2* and a trending decrease in *Tgfb1* in HFD-fed mice (Supplementary Fig. 2m). Furthermore, similar to the colon, HFD feeding decreased the frequency of CX3CR1^+^ macrophages in the MLN and trended to decrease the proportion of their CD11b^+^ CD11c^+^ subset (Supplementary Fig. 2n, o). While total DCs were not altered in the MLN, small differences were seen in the CD103^+^ CD11b^−^ and CD103^−^ CD11b^+^ subsets in HFD-fed mice (Supplementary Fig. 2p, q). Overall, these results demonstrate that the compromised intestinal production of IgA is associated with HFD-induced reduction in cellular and secreted immune mediators involved in IgA CSR and production.

### IgA deficiency worsens glucose homeostasis during HFD

**Fig. 3:**
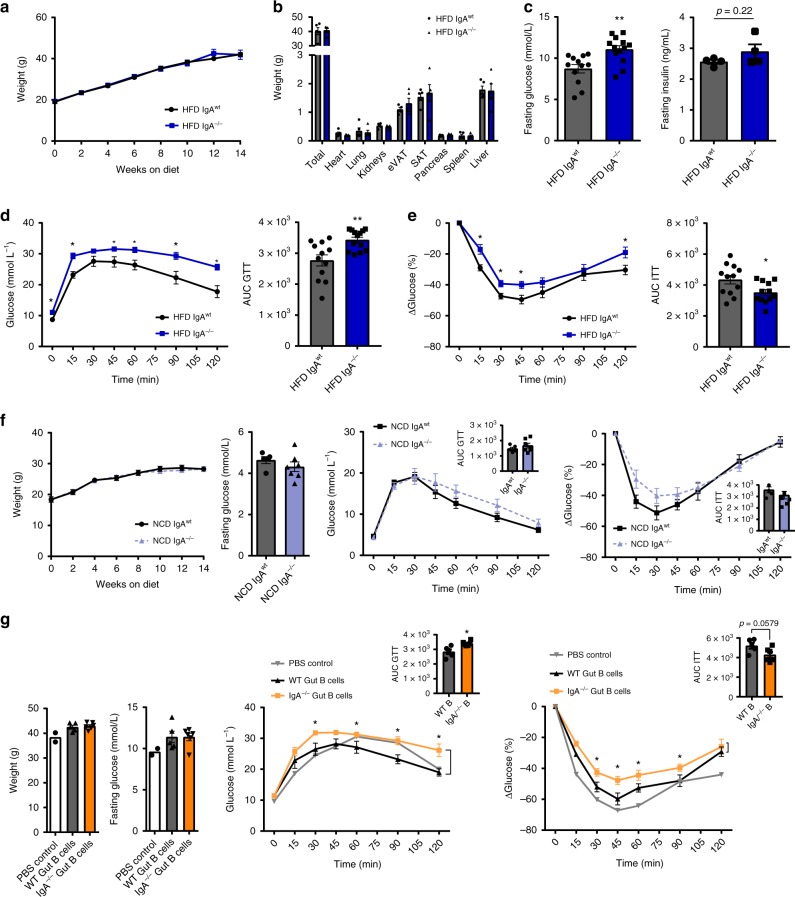
Immunoglobulin A (IgA) deficiency, specifically in intestinal B cells, increases insulin resistance in high fat diet (HFD)-fed mice. **a** Weight gain (*n* = 21 WT, 16 IgA^−/−^ HFD; 4 distinct cohorts) and **b** organ weights (*n* = 5 WT, 5 IgA^−/−^) of IgA^−/−^ mice fed HFD compared to wild-type (WT) littermate controls over 14 weeks of diet. **c** Fasting glucose (left), fasting insulin (right) (*n* = 4 WT, 4 IgA^−/−^), **d** glucose tolerance test (GTT) (left) also represented by area under the curve (AUC) (right), and **e** insulin tolerance test (ITT) (left) also represented by AUC (right) in IgA^−/−^ mice fed HFD (*n* = 12 WT, 12–13 IgA^−/−^, 3 distinct cohorts). **f** Weight gain (left) (*n* = 5 WT, 8 IgA^−/−^ NCD), fasting glucose (middle left), glucose tolerance also represented by AUC (middle right), and insulin tolerance and AUC (right) of IgA^−/−^ mice fed normal control diet (NCD) compared to WT littermate controls after 14 weeks of diet (*n* = 5 WT, 7 IgA^−/−^ NCD). **g** Weights (left), fasting glucose levels (left middle), blood concentrations of glucose during GTT and AUC (right middle), and ITT and AUC (right) of 12-week HFD-fed B cell-deficient muMT^−^ (B^null^) mice 2 weeks post adoptive transfer of IgA^−/−^ or WT intestinal pan B cells, or a sham phosphate-buffered saline (PBS) control (*n* = 2 PBS control, 5 WT gut B cells, 6 IgA^−/−^ gut B cells). Data are means ± SEM. * denotes *p* < 0.05 and ** denotes *p* < 0.01

Given that IgA^+^ B cells and plasma cells within the intestine were primarily affected by HFD feeding, we next sought to determine a role for IgA in obesity and IR. IgA-deficient (*Igha*^−/−^, referred to hereafter as IgA^−/−^) mice and littermate control wild-type (IgA^wt^ or WT) mice were fed a HFD or NCD for 14 weeks and then assessed for metabolic parameters. Of note, IgA^−/−^ mice show normal organ and immune system development^37^. During HFD feeding, IgA^−/−^ mice did not show differences in body weight gain (Fig. 3a) or energy metabolism, compared to WT controls (Supplementary Fig. 3a). Organ weights were similar between HFD-fed IgA^−/−^ and WT controls (Fig. 3b). Histological analysis of the ileum and colon did not show differences in tissue morphology between HFD-fed IgA^−/−^ and WT controls (Supplementary Fig. 3b). However, we observed a mild qualitative histological increase in macrovesicular steatosis within the livers of IgA^−/−^ mice fed a HFD for 14 weeks (Supplementary Fig. 3c). To evaluate glucose metabolism in HFD-fed IgA^−/−^ mice, we performed glucose tolerance (GTTs) and insulin tolerance tests (ITTs) after 14 weeks of feeding. HFD-fed IgA^−/−^ mice displayed increased fasting glucose levels (Fig. 3c), as well as worsened glucose (Fig. 3d) and insulin tolerance (Fig. 3e). To determine if the effects of IgA on glucose metabolism is specific to HFD feeding, we fed IgA^−/−^ and littermate control mice a NCD for 14 weeks prior to assessment of metabolic parameters. NCD-fed IgA^−/−^ mice showed no differences in weight gain, energy, or glucose metabolism, suggesting that IgA regulates glucose metabolism specifically in response to HFD feeding (Fig. 3f, Supplementary Fig. 3d).

We next determined whether B cells and plasma cells contribute to the changes in glucose metabolism observed in IgA^−/−^ mice. As IgA is most commonly found within the gastrointestinal and mucosal immune system, we assessed if IgA-deficient B cells, specifically in the gut and its associated lymphoid system, are responsible for worsened metabolic disease. We adoptively transferred pan B cells (includes plasma cells) enriched from either IgA^−/−^ or WT intestines pooled from the ileum, colon, Peyer’s patches, and MLN into B cell-deficient (muMt^−^) mice fed a HFD for 14 weeks. Transferred B and plasma cells preferentially reconstituted in the colon, compared to small intestine and lymphoid organs including PP, MLN, and spleen (Supplementary Fig. 3e). Two weeks post transfer, despite no differences in body weight and fasting glucose, the B cell-deficient mice that received intestinal pan B cells from IgA^−/−^ mice showed worsened glucose and insulin tolerance compared to mice that received intestinal pan B cells from WT controls (Fig. 3g). Our data suggest that IgA derived from intestinal B cells potentially play a critical role in whole-body glucose homeostasis.

### Loss of IgA exacerbates gut and VAT inflammation with DIO

**Fig. 4:**
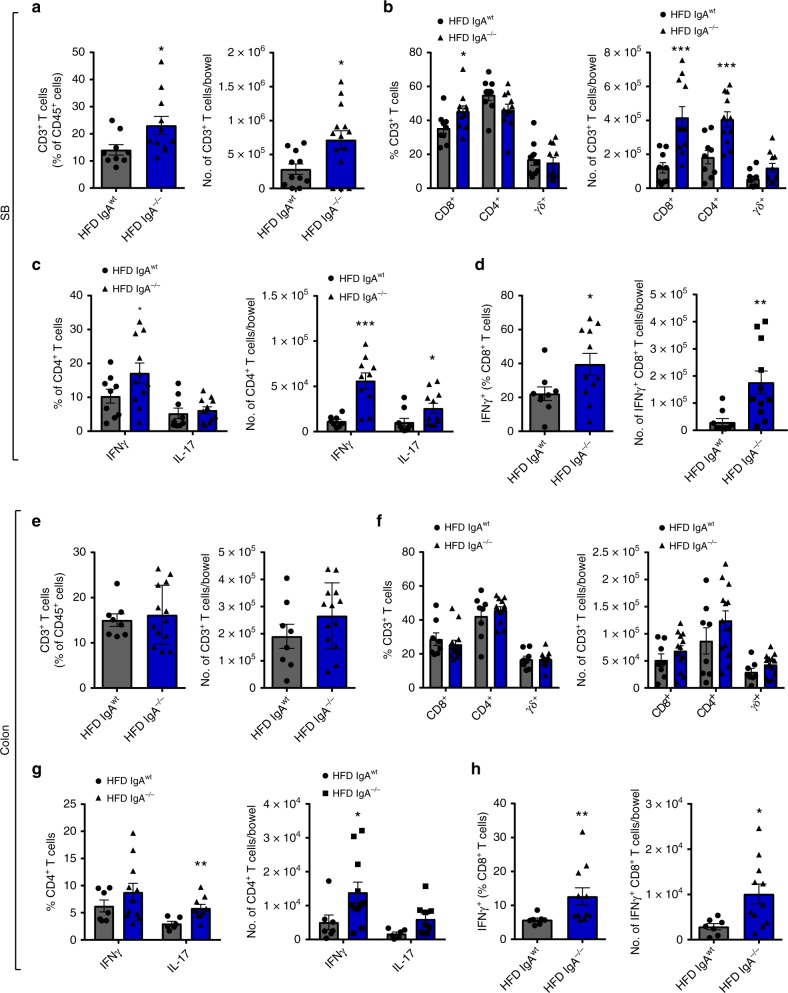
Loss of immunoglobulin A (IgA) promotes pro-inflammatory intestinal T cell responses in high fat diet (HFD)-fed mice. Frequency (left) and absolute number (right) of **a** CD3^+^ T cells, **b** T cell subsets, **c** interferon-γ (IFNγ) and interleukin-17 (IL-17)-producing (T-helper type 1 (Th1) and Th17, respectively) CD4^+^ T cells, **d** IFNγ-producing CD8^+^ T cells in the distal small intestinal lamina propria (LP) of HFD-fed IgA^−/−^ compared to wild-type (WT) controls (*n* = 9 WT, 11 IgA^−/−^, 4 experiments). **e** Frequency (left) and absolute number (right) of CD3^+^ T cells, **f** T cell subsets, **g** IFNγ- and IL-17-producing (Th1 and Th17, respectively) CD4^+^ T cells, **h** IFNγ-producing CD8^+^ T cells in colon LP of HFD-fed IgA^−/−^ compared to WT controls (*n* = 7–8 WT, 11–13 IgA^−/−^, 4 experiments). Data are means ± SEM. * denotes *p* < 0.05, ** denotes *p* < 0.01, and *** denotes *p* < 0.001

Low-grade chronic intestinal inflammatory changes mediated by local changes in T cell populations have been shown to be an important player in HFD-induced obesity and IR^7–9^. We next assessed T cell immune populations within the distal small bowel and colon of IgA^−/−^ mice after 14 weeks of HFD feeding. Within the small bowel LP, the percentage and number of CD3^+^ T cells increased in HFD-fed IgA^−/−^ mice, compared to HFD-fed IgA^wt^ controls (Fig. 4a). Within the T cell compartment, we found an increased percentage and number of CD8^+^ T cells and augmented number, but not percentage, of CD4^+^ T cells (Fig. 4b). Intracellular staining of pro-inflammatory cytokines showed that IFNγ-producing CD4^+^ (Fig. 4c) and CD8^+^ T cells (Fig. 4d) were increased in the distal small intestine in HFD-fed IgA^−/−^ mice. While no differences were detected in CD3^+^ T cells (Fig. 4e) or its CD4^+^, CD8^+^, and γδ^+^ fractions in the colon (Fig. 4f), we observed increases in the number of IFNγ-producing CD4^+^ and CD8^+^ T cells, as well as in the percentage of IL-17-producing CD4^+^ T cells (Fig. 4g, h). Percentages of γδ^+^ T cell subsets did not differ in IgA deficiency in either the small bowel or colon, but IFNγ-producing γδ^+^ T cells were increased in numbers within the small bowel (Supplementary Fig. 4a, b). In addition, the percentage of Foxp3^+^ CD4^+^ Tregs in the small bowel and colon were unchanged in IgA^−/−^ mice, although numbers were again increased in the small bowel (Supplementary Fig. 4c), but not in the colon (Supplementary Fig. 4d). Changes to T cell inflammatory tone in the small intestine and colon were not observed in NCD-fed IgA^−/−^ mice compared to WT controls (Supplementary Fig. 5a–l). Thus, IgA deficiency during HFD feeding leads to changes in intestinal inflammatory potential in both the small bowel and colon, which may contribute to the overall worsening of metabolic disease.

**Fig. 5:**
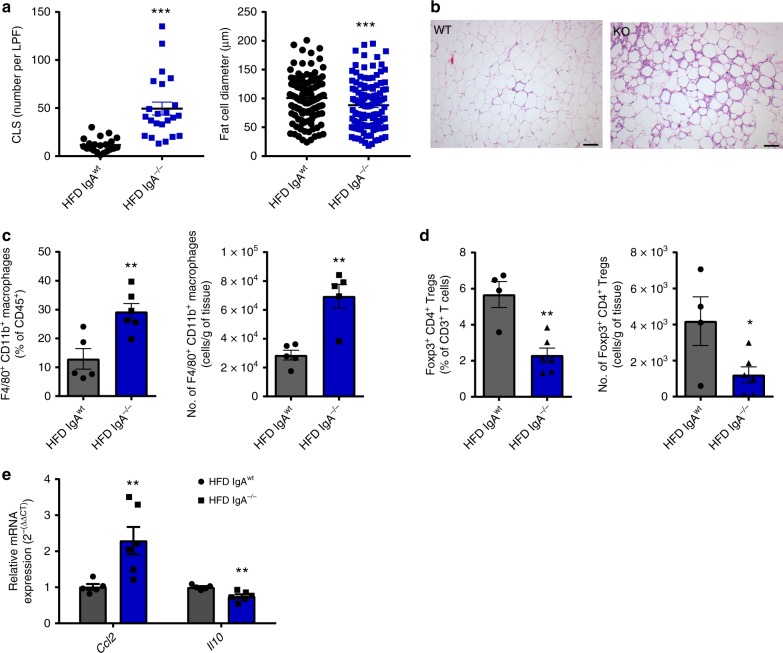
Immunoglobulin A (IgA) deficiency exacerbates immune-cell-mediated visceral adipose tissue (VAT) inflammation. **a** Enumeration of crown-like structures (CLS) per ×100 low power field (LPF) (left) and fat cell diameter (right) within the VAT of high fat diet (HFD)-fed WT and IgA^−/−^ after 14 weeks of diet (*n* = 3 mice/group, 10–12 LPF per mouse for CLS, 120–129 cells from three mice for cell diameter). **b** Representative histological images of VAT of HFD-fed WT (left) and IgA^−/−^ (right) after 14 weeks of diet (scale bar =100 µm). **c** Percentage (left) and absolute number (right) of total CD11b^+^ F4/80^+^ macrophages within the VAT stromal vascular fraction measured by flow cytometry (*n* = 5–6 pooled mice, three experiments). **d** Frequency (left) and absolute number (right) of Foxp3^+^ CD4^+^ regulatory T cells (Tregs) in the stromal vascular cells of VAT in IgA^−/−^ mice fed HFD for 14 weeks compared to WT controls (*n* = 4 WT, 6 IgA^−/−^ pooled mice; 4 experiments). **e** Relative mRNA expression levels of CCL2 and interleukin-10 (IL-10) within VAT of HFD fed WT and IgA^−/−^ mice (*n* = 5 WT, 6 IgA^−/−^, 2 experiments). Data are means ± SEM. * denotes *p* < 0.05, ** denotes *p* < 0.01, and *** denotes *p* < 0.001

Changes in the inflammatory potential of the intestinal immune system are thought to prime downstream inflammation in VAT, a major driver of obesity-related IR. Compared with HFD-fed littermate controls, IgA^−/−^ mice had a large number of crown-like structures (CLS) in the VAT, which are indicative of macrophages surrounding dying adipocytes (Fig. 5a, left, and 5b). The increase in CLS around adipocytes minimally reduced the size of adipocytes in IgA^−/−^ mice compared to WT controls (Fig. 5a, right, 5b). In support of increased CLS observed inside VAT, we assessed changes in total macrophage populations within the VAT of IgA^−/−^ mice and WT controls. Indeed, IgA^−/−^ mice had increased percentages and numbers of F4/80^+^ CD11b^+^ VAT macrophages, compared to WT mice (Fig. 5c). However, M1-like (CD11c^+^ CD206^−^) and M2-like (CD11c^−^ CD206^+^) macrophage subsets and the macrophage activation markers CD80 and CD86 did not differ with IgA deficiency (Supplementary Fig. 6a, b). Interestingly, we observed decreased Tregs within the VAT of IgA^−/−^ mice fed a HFD for 14 weeks, which is associated with aggravated inflammation and IR (Fig. 5d). Changes in the percentage and number of other T cell populations were unaltered inside the VAT (Supplementary Fig. 6c–g). Consistent with these findings we observed an increase in the gene expression of monocyte chemoattractant CCL2 and pro-inflammatory cytokine IL-6, as well as a decrease in the expression of the anti-inflammatory cytokine IL-10 in the VAT of obese IgA^−/−^ mice (Fig. 5e and Supplementary Fig. 6h). Overall, these findings suggest a shift to increased immune-cell-mediated inflammation within the VAT of IgA^−/−^ HFD-fed mice.

### IgA controls host–microbiome homeostasis during DIO

**Fig. 6:**
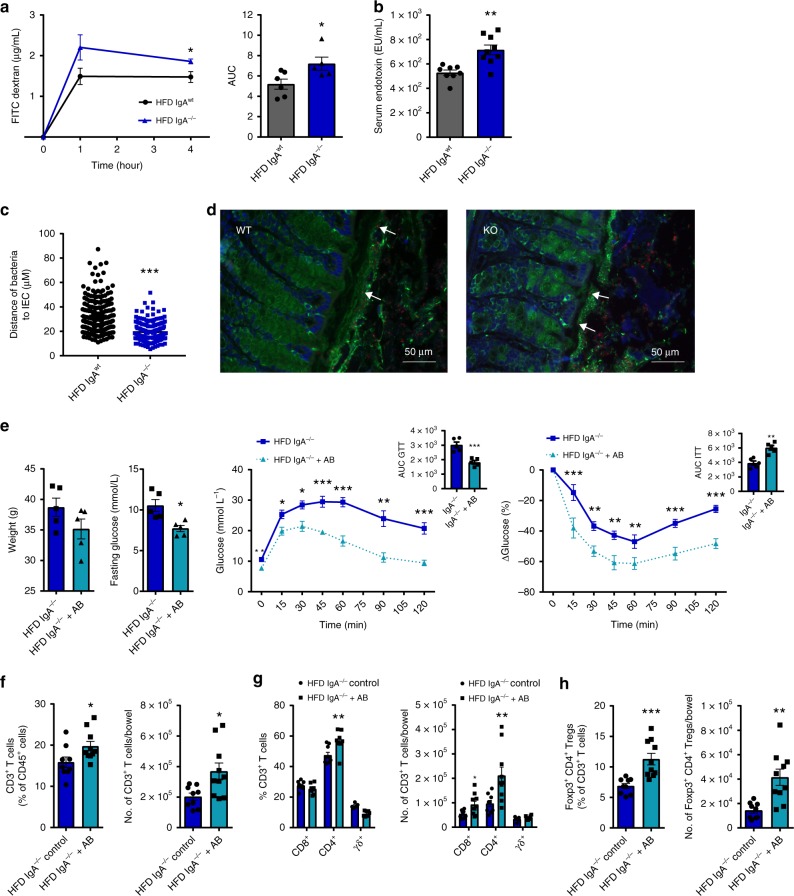
Altered glucose metabolism in high fat diet (HFD)-fed immunoglobulin A (IgA)-deficient mice is linked to host–microbe interactions. **a** Intestinal permeability assay measuring concentration of fluorescein isothiocyanate (FITC)-labelled-dextran by fluorescence (left) and represented by area under the curve (AUC) (right) in collected plasma 1 and 4 h post gavage in HFD-fed wild-type (WT) and IgA^−/−^ mice (*n* = 6 WT, 5 IgA^−/−^ mice). **b** Serum endotoxin levels in WT and IgA^−/−^ mice fed HFD for 14 weeks (*n* = 8 WT, 9 IgA^−/−^ mice). **c** Distance of bacteria from intestinal epithelial cells (IECs) in HFD-fed WT and IgA^−/−^ mice (*n* = 4/group). **d** Representative images of fluorescence in situ hybridization (FISH) and immunofluorescence staining of the bacteria (red), mucous layer (green), and intestinal cells (4′,6-diamidino-2-phenylindole (DAPI)-blue) of colon (scale bar = 50 µm; white arrows indicate representative encroaching bacteria to intestinal epithelium). **e** Weights (left), fasting glucose levels (left middle), blood glucose concentrations with AUCs during glucose tolerance (GTT) (right middle) and insulin tolerance test (ITT) (right) in HFD-fed IgA^−/−^ mice treated with antibiotics in drinking water for 4 weeks compared to HFD-fed IgA^−/−^ untreated controls (*n* = 5 mice/group). Frequency (left) and absolute number (right) of **f** CD3^+^ T cells, **g** T cell subsets, and **h** Foxp3^+^ CD4^+^ Tregs within the colonic lamina propria (LP) in IgA^−/−^ mice treated with antibiotics in drinking water for 4 weeks compared to IgA^−/−^ untreated controls fed HFD for total of 14 weeks (*n* = 9 WT, 10 IgA^−/−^ mice, 2 experiments). Data are means ± SEM. * denotes *p* < 0.05, ** denotes *p* < 0.01, and *** denotes *p* < 0.001

In addition to a shift towards an inflammatory profile, obesity-related IR is associated with dysbiosis, increased intestinal permeability, and metabolic endotoxemia. Given the low-grade inflammatory changes identified in the intestine of HFD fed IgA^−/−^ mice, and the role of IgA in maintaining intestinal host–microbiome homeostasis, we also investigated whether IgA deficiency predisposes to altered intestinal permeability and microbial encroachment. Indeed, HFD-fed IgA^−/−^ mice had worsened intestinal permeability as demonstrated by an FD4 intestinal paracellular permeability assay (Fig. 6a) and exhibited higher levels of serum endotoxins compared to HFD-fed WT controls (Fig. 6b). In contrast, NCD-fed IgA^−/−^ mice displayed no significant differences in intestinal permeability or serum endotoxin levels compared to NCD-fed WT mice (Supplementary Fig. 7a, b). In corroboration with these findings, using fluorescence in situ hybridization (FISH) and immunofluorescence staining for bacterial detection  in the colon, we further observed that the microbiota of HFD-fed IgA^−/−^ mice displayed worsened encroachment^38^ to host cells as demonstrated by the reduced distance of bacteria to intestinal epithelial cells (IECs) (Fig. 6c, d). Together, increased intestinal permeability, microbial encroachment, and higher serum endotoxin levels likely contribute to the overall increased inflammatory profile within the intestine and VAT, propelling metabolic disease.

**Fig. 7:**
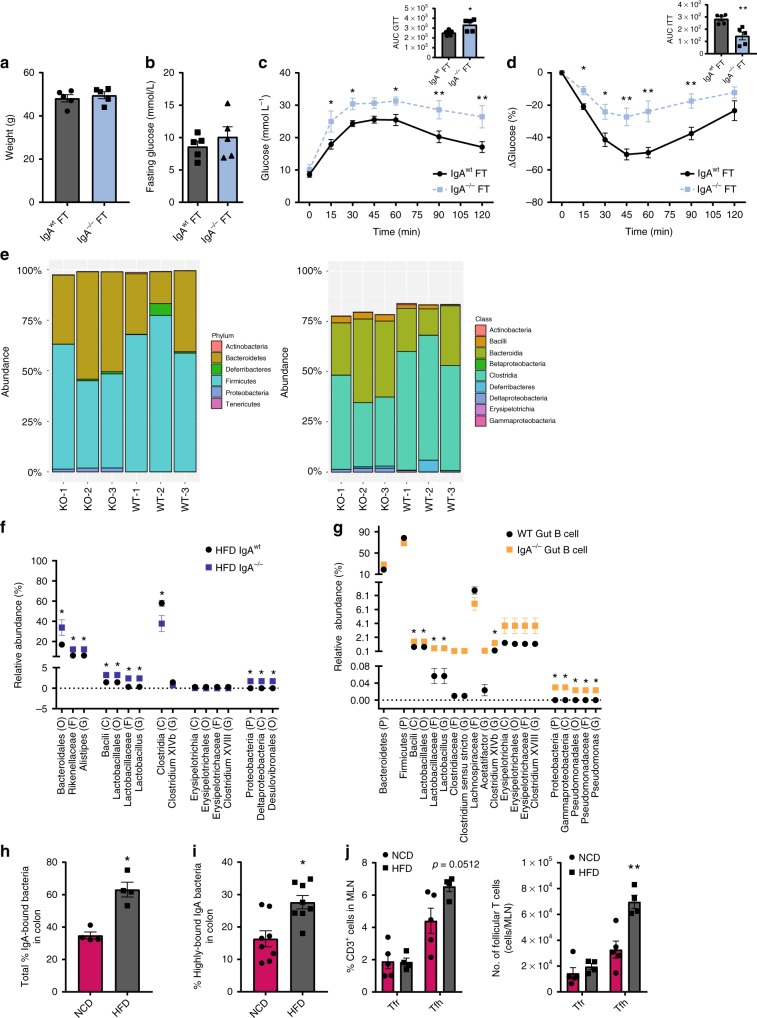
Immunoglobulin A (IgA) deficiency promotes host bacteria pathogenicity during diet-induced obesity (DIO). **a** Body weight, **b** fasting glucose, **c** glucose tolerance (also represented by area under the curve (AUC)), and **d** insulin tolerance (also represented by AUC) of antibiotic-treated HFD-fed wild-type (WT) mice transplanted with fecal matter from HFD-fed IgA^−/−^ and IgA^wt^ mice (*n* = 5/group). **e**–**g** 16S rRNA sequencing of gut microbial populations within the distal small intestine, colon (without cecum), and cecum alone of IgA^−/−^ and WT mice after 14 weeks of HFD feeding and within colons of intestinal B cell adoptive transfer HFD-fed muMT^−^ (B^null^) mice 2 weeks post transfer. **e** Relative abundances of bacterial communities at the phylum (left) and class (right) levels in the colon of HFD-fed IgA^−/−^ (knockout (KO)) and WT mice (*n* = 3/group). **f** Significant bacterial taxa of the colonic stool in IgA^−/−^ compared to WT HFD-fed mice (*n* = 3/group; multiple statistical testing corrected with false discovery rate (FDR) Benjamini and Hochberg approach). **g** Significant bacterial taxa with colonic stool of HFD-fed B cell-deficient muMT^−^ mice adoptively transferred with either IgA^−/−^ or WT intestinal pan B cells (*n* = 3/group; multiple statistical testing performed with FDR Benjamini and Hochberg approach). **h** Percentage of total (*n* = 4/group) and **i** high-affinity IgA-bound bacteria within the colonic contents of HFD-fed mice compared to NCD controls (*n* = 8/group, 2 experiments). **j** Frequency (left) and absolute numbers (right) of T follicular regulatory (Tfr) and T follicular helper (Tfh) CD3^+^ T cells in the colon draining  mesenteric lymph nodes (*n* = 5 WT, 4 IgA^−/−^). Data are means ± SEM. *p < 0.05 and ** denotes *p* < 0.01

We next determined if the effects of IgA on glucose metabolism are regulated by the gut microbiota. First, we eliminated the intestinal microbiota using 4 weeks of broad-spectrum antibiotics treatment^5^ in IgA^−/−^ mice fed HFD for 10 weeks and assessed the metabolic parameters. After 4 weeks of antibiotic treatment, we confirmed a reduction in fecal bacterial load in antibiotic-treated compared to untreated HFD-fed IgA^−/−^ mice via 16S quantitative PCR (qPCR) (Supplementary Fig. 7c). Antibiotic-treated HFD-fed IgA^−/−^ mice exhibited lower fasting glucose levels, and improved glucose and insulin tolerance (shown over time and by area under the curve) without significantly altering body weight compared to untreated HFD-fed IgA^−/−^ controls (Fig. 6e). Although broad increases in multiple T cell populations were noted within the colon (Fig. 6f, g), antibiotic-treated IgA^−/−^ mice displayed a higher percentage and number of anti-inflammatory Tregs in the colon (Fig. 6h), consistent with the improved metabolic phenotype. The increase in Tregs was mainly seen in the colon and not in the small bowel (Supplementary Fig. 7d, e) or VAT (Supplementary Fig. 7f, g). To provide further evidence for a pathogenic role of the gut microbiota in contributing to worsened metabolic parameters observed in IgA^−/−^ mice, we hypothesized that fecal microbiota transplantation of HFD-fed IgA^−/−^ mice into gut microbiota-depleted mice would recapitulate the metabolic disease phenotype. To test this concept, we transferred colonic fecal material from HFD-fed IgA^−/−^ and WT mice into HFD-fed WT mice subjected to an intensive antibiotic regimen (AB) or into HFD-fed germ-free (GF) mice and then assessed for glucose and insulin tolerance after 4 weeks. Consistent with microbial transfer, we observed increased fecal bacterial load from colonic stool of AB and GF mice recipients transplanted with HFD-fed IgA^−/−^ or IgA^wt^ fecal matter via 16S qPCR (Supplementary Fig. 8b). While no differences in fasting glucose or weights were observed, both AB and GF mice displayed worsened glucose intolerance and IR when engrafted with microbiota from HFD-fed IgA^−/−^ mice compared to HFD-fed WT microbiota (Fig. 7a–d and Supplementary Fig. 8a). Together, these data suggest that the gut microbiota contributes to the exacerbated metabolic phenotype observed in IgA deficiency during HFD feeding.

Given a role for the microbiota in mediating effects of IgA deficiency during HFD feeding, we investigated the changes to the microbial taxa in HFD-fed IgA^−/−^ mice compared to WT controls. More specifically, we determined if IgA deficiency predisposes to potentially pathogenic organisms linked to obesity and/or IR. Using 16S rRNA sequencing, we identified a higher abundance of Proteobacteria at the phylum level residing in the colons of IgA^−/−^ mice fed a HFD for 14 weeks compared to WT controls (Fig. 7e, left). In this phylum, bacteria belonging to the class *Deltaproteobacteria* and order *Desulfovibrionales* were increased (Fig. 7e, right, 7f). In addition, the HFD-fed IgA^−/−^ mice harbored more abundantly *Lactobacillus* and *Alistipes* and reduced amounts of bacteria from the *Clostridia* class compared to HFD-fed WT mice (Fig. 7f). Linear discriminant analysis effect size (LEfSe) analysis of the colonic microbiota also exhibited additional bacterial taxa differing between IgA^−/−^ and WT mice (Supplementary Fig. 8c).

To assess if intestinal IgA-producing B cells can directly dictate the microbiota in the setting of HFD feeding, stool from our pan B cell transfer experiments (Fig. 3g) was sequenced with 16S rRNA sequencing. Remarkably, B cell-deficient (muMT^−^) recipients of the adoptively transferred intestinal pan B cells of IgA^−/−^ mice harbored greater abundance of similar bacterial taxa observed in mice with global IgA deficiency, including Proteobacteria within the colon (Fig. 7g), suggesting that intestinal IgA B cell populations can imprint distinct microbial compositions of some taxa during HFD feeding. The recipients also had greater relative abundances of *Lactobacillus*, *Clostridium XIVb*, and *Pseudomonas* compared to recipients of WT gut pan B cells. Other differences between certain bacterial taxa in the adoptively transferred B cell-deficient mice were seen by LEfSe analysis (Supplementary Fig. 8d). Principal coordinate analysis did not exhibit significant distances between the IgA^−/−^ and WT microbiota in various locations within the intestine (small intestine, cecum, and colon) (Supplementary Fig. 8e). IgA deficiency trended towards reduced α-diversity of the bacterial communities within the distal small bowel and colon (distal to cecum) and did not alter diversity in the cecum of HFD-fed mice or in the adoptively transferred B cell-deficient mice (Supplementary Fig. 8f). Overall, IgA deficiency, specifically in the intestine, can lead to altered abundance or growth of bacterial taxa during obesity, including those of the Proteobacteria phylum.

Since IgA can directly interact with bacteria through microbial binding, we examined the quality of the IgA response to the microbiota during HFD feeding in WT mice. Consistent with the notion of DIO promoting the presence of more pathogenic, and potentially penetrant microbes, we observed that within colonic stool, HFD feeding induced an increase in the proportion of bacteria bound by IgA (Fig. 7h) as well as an increase in the percentage of bacteria bound to IgA with high affinity (Fig. 7i). Similar increases were observed in the small intestinal IgA-bound bacteria (Supplementary Fig. 8g). As follicular T cells are crucial for regulating the affinity and polyreactivity of IgA, we examined their presence in the MLN draining the colon^39^. HFD feeding resulted in increased numbers of high-affinity-promoting follicular T helper (Tfh) cells with no alterations to the amounts of regulatory T follicular cells (Fig. 7j). Overall, these findings suggest that during HFD feeding, IgA responds to the increased burden of pathogenic and potentially penetrant microbial species within the intestine.

### Glucose-lowering therapies modify intestinal IgA

**Fig. 8:**
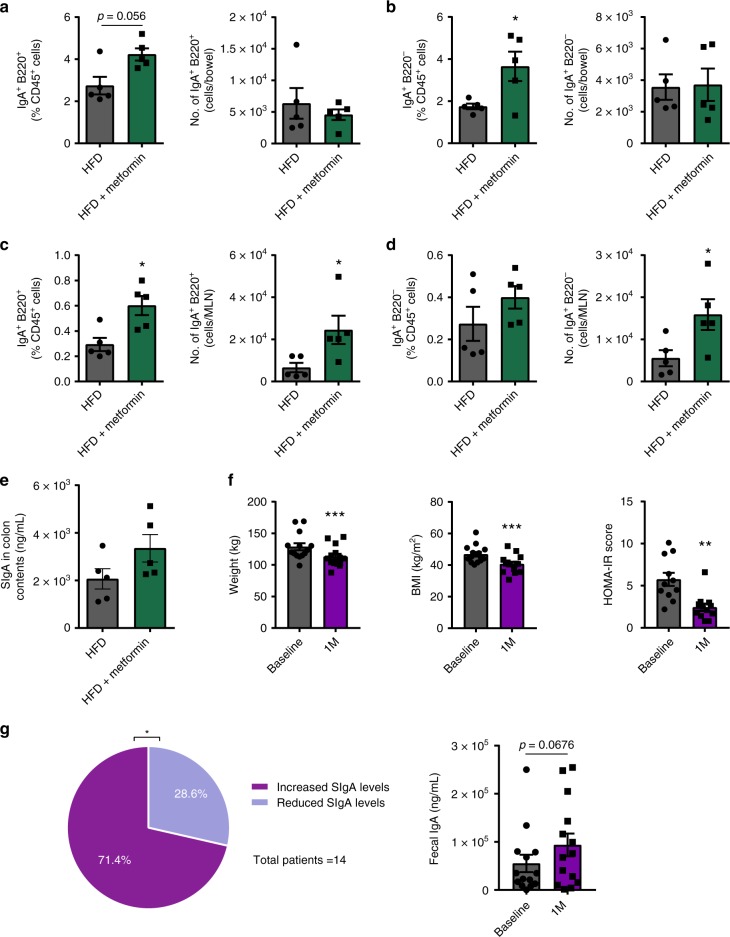
Metformin treatment and bariatric surgery interventions can manipulate intestinal immunoglobulin A (IgA). Frequency and absolute numbers of IgA-producing **a** B cells (B220^+^) and **b** plasma cells (B220^−^) in the colon of high fat diet-fed mice treated with or without metformin in drinking water (300 mg kg^−1^ per day) for 14 weeks (*n* = 5/group). Frequency and absolute numbers of IgA-producing **c** B cells (B220^+^) and **d** plasma cells (B220^−^) in the colon draining mesenteric lymph node (MLN) (*n* = 5/group). **e** Colonic secretory IgA (SIgA) levels in HFD-fed mice treated with or without metformin in drinking water for 14 weeks (*p* = 0.1). **f** Weight (left), body mass index (BMI) (middle), and homeostatic model assessment-insulin resistance (HOMA-IR) score (right) of obese patients at baseline and 1 month post-bariatric surgery (*n* = 14 patients; Wilcoxon’s matched-pairs test). **g** Left: Representative pie chart demonstrating number of patients with increased vs. decreased fecal IgA 1 month post-bariatric surgery (*p* = 0.02 *χ*^2^chi-square test) and Right: total fecal IgA levels at baseline and one month post surgery (*n* = 14 patients, *p* = 0.0676 Wilcoxon’s matched-pairs test). Data are means ± SEM. * denotes *p* < 0.05, ** denotes *p* < 0.01, and *** denotes *p* < 0.001

Metformin is one of the most commonly prescribed treatments for type 2 diabetes; however, its mechanism of action is not fully understood. Recently, the gut microbiota has been proposed as a major mechanism for metformin’s therapeutic action^40–42^, but its effects on the intestinal immune system remains unknown. We investigated whether metformin treatment (300 mgkg^−1^ per day) in drinking water of HFD-fed mice can impact intestinal immunity and local IgA levels compared to mice fed HFD alone. After 14 weeks of diet, metformin treatment prevented the reduction of IgA-producing cells previously seen in the intestine of HFD-fed mice. Specifically, percentages, but not the absolute number, of IgA^+^ B and plasma cells were increased in the colon LP (Fig. 8a, b) while both the percentage and number of IgA^+^ B cells were increased in MLNs of metformin-treated mice (Fig. 8c). The number of IgA^+^ plasma cells was also increased in the MLN (Fig. 8d). In addition, levels of SIgA trended to increase in the colonic fecal contents of metformin-treated mice compared to HFD alone (Fig. 8e). Metformin did not influence the amounts of IgA^+^ cells in the small bowel or PPs (Supplementary Fig. 9a, b) or Tregs in the small bowel, colon, or MLNs (Supplementary Fig. 9c–e). Intestinal permeability was also improved in metformin-treated mice during an FD4 (fluorescein isothiocyanate-dextran) assay (Supplementary Fig. 9f).

To translate our findings into humans, we next determined whether intestinal IgA content is altered by a therapeutic intervention for metabolic disease in humans, such as bariatric surgery. We analyzed the fecal SIgA content of 14 patients at baseline and 1-month post-bariatric surgery, during which metabolic improvements were seen in weight, body mass index, and homeostatic model assessment-insulin resistance (Fig. 8f and Supplementary Table 1). Interestingly, we observed that in a significant proportion (10 out of 14, or 71%) of these patients, there were increases in SIgA levels 1 month post surgery (Fig. 8g, left). Consistently, we noted that overall fecal SIgA levels within the patient cohort trended towards an increase 1 month post surgery (Fig. 8g, right). Taken together, we have demonstrated that both pharmacological and surgical intervention regimens of T2D have the potential to influence IgA levels within the intestine, further supporting IgA as a regulator of glucose homeostasis.

## Discussion

The intestinal immune system is emerging as an important player in obesity-related IR^1,7,9,43,44^. While changes in intestinal IgA have been previously linked to intestinal diseases such as inflammatory bowel disease and colitis-associated cancers^45,46^, it was unknown if SIgA and IgA-producing cells are altered in the intestine during DIO. Here, we demonstrate a reduction of intestinal IgA during obesity and the downstream implications of the loss of IgA on glucose homeostasis. It is likely that changes to the selection of IgA in addition to the reduced production of this immunoglobulin within the colon specifically impact intestinal homeostasis during obesity. Examination of mechanisms potentially responsible for diminished total colonic fecal IgA in obesity revealed a site-specific loss in factors promoting IgA production, such as TGF-β1 and IL-5, along with decreased expression of enzymes required for the synthesis of RA. Intestinal DCs and macrophages express these enzymes to synthesize RA and also secrete the aforementioned factors, which can imprint gut homing of IgA B cells, as well as the synthesis and secretion of IgA^16^. Our analyses revealed changes in DC and macrophage subsets that have been previously linked to altered B cell function and pro-inflammatory skewing of intestinal T cells^32–35^.

Given the previously described reduced frequency of NKp46^+^ CD4^−^ ILCs in the colon of obese mice^8^, it remains to be determined whether levels of intestinal ILC-produced Csf2 are altered during HFD feeding, which may also partially explain changes observed in IgA-producing cells and IgA-promoting mediators^47^. Manipulating these intestinal immune cell populations and/or their secreted factors, such as via administration of all-*trans*-RA, might serve as a therapeutic avenue for obesity-associated metabolic dysfunction by enhancing IgA presence in the colon to curb the pathogenic consequences of a dysbiotic penetrant microbiota^47–49^.

We further demonstrated that IgA is a key regulator of glucose metabolism as obese IgA-deficient mice developed worsened glucose tolerance and insulin sensitivity, independent of weight differences. These changes were not seen during NCD, suggesting that IgA is influenced by HFD-associated changes such as in the gut microbiota and intestinal inflammatory tone. Indeed, IgA^−/−^ during HFD feeding exhibit a greater pro-inflammatory shift in immune cells compared to WT controls. It is important to note that although genetic deficiency of IgA does not result in abnormal immune system development, these mice have been reported to have increased serum IgM and IgG^37^, which may potentially impact glucose regulation^4,18^. However, it is unlikely that this caveat plays a major role in dictating whole-body glucose in IgA deficiency as we and others have shown HFD-induced local intestinal immune changes to control VAT and systemic inflammation through intestinal barrier modulation^7,8,13^, and the strong influence of the gut microbiota in controlling metabolism in IgA deficiency revealed in the current study.

In addition to an altered intestinal inflammatory tone, we observed an increase in immune-cell-mediated inflammation in the VAT, a primary tissue involved in metabolism-related inflammation and IR^2^. The VAT of HFD-fed IgA^−/−^ mice contained less Tregs and higher accumulation of macrophages and CLS, a hallmark of VAT inflammation during obesity. There is a possibility that luminal antigens or immune cells from the intestine may deposit in other tissues such as VAT and liver to signal local inflammatory changes. Consistently, we noted increased CCL2 levels inside the VAT of HFD-fed IgA^−/−^ mice, which may be linked to increased bacterial product translocation seen in IgA^−/−^ mice^50^. However, we cannot neglect the possible contribution of IgA loss directly within the VAT and other metabolic organs to promote inflammation, although the relative amount of IgA present within these tissues is much less compared to mucosal sites.

Under homeostatic conditions, IgA functions to modulate the gut microbiota and prevent the binding of bacteria and microbial products to the intestinal epithelium^51^. Several studies have shown that HFD-triggered gut dysbiosis and gut inflammation damages the intestinal wall increasing intestinal permeability and metabolic endotoxemia^6,8^. Here, we demonstrated that compared to HFD-fed WT mice, HFD-fed IgA^−/−^ mice have increased intestinal permeability, similar to polymeric Ig receptor (pIgR)^−/−^ mice that cannot transport IgA into the intestinal lumen^52,53^, which may also be linked to worsened bacterial encroachment and pro-inflammatory cytokine-mediated disruption of epithelial tight junctions^6,8^. Future investigation can also examine the ability of the penetrating bacteria to trigger systemic responses including metabolic endotoxemia by analyzing, for example, bacteria-specific IgG responses in the serum.

We show that IgA loss leads to changes to intestinal microbial composition in the setting of obesity and that the metabolic phenotype in HFD-fed IgA^−/−^ mice can be conferred to microbiota-deficient mice through fecal transplantations. IgA^−/−^ in our colony was associated with increases in bacteria of the Proteobacteria phylum, which include *Deltaproteobacteria*, and other taxa including *Lactobacillus* and *Alistipes*. Proteobacteria have been previously linked to increased gut inflammatory tone and obesity in mice and humans^54^. It is thought that during dysbiosis, an unstable microbial community allows for the abnormal expansion of potentially opportunistic bacteria such as Proteobacteria. The increased presence of Proteobacteria in mice that received IgA-deficient intestinal B cells suggests that B cells, specifically IgA, can affect microbial composition. Perhaps, the removal of IgA from the host–microbe ecosystem adds another level of destabilization in the bacterial community and furthers dysbiosis induced by HFD or obesity. Moreover, several groups have shown high-affinity IgA coating of gut bacteria to be directly related to the pathogenesis of several gut-related diseases such as colitis and malnutrition, and can be used for the manipulation of the gut microbiota to ameliorate disease^55–57^. In accordance with these observations, we found that HFD feeding also promoted the presence of higher affinity IgA in the colon, likely due to increased presence of penetrative pathogenic bacteria. As such, the role of T-dependent and -independent signals related to the regulation of IgA and IgA affinity during DIO warrants future investigation. We further demonstrated that eliminating the gut microbiota with antibiotics in HFD-fed IgA^−/−^ mice improved glucose homeostasis. Taken together, these data suggest that alterations to the gut microbiota during IgA deficiency are a plausible mechanism behind worsened intestinal inflammation and disease.

Lastly, we identified that intestinal IgA levels can be manipulated with pharmacological and surgical intervention regimens for glucose homeostasis. Treatment with metformin in obese mice increases IgA-producing immune cells, potentially raises SIgA levels, and improves intestinal permeability. Further studies are needed to determine if these changes occur as a result of metformin action on gut bacteria or a direct effect on AMPK activity in intestinal immune populations. It has been previously noted that the gut microbiota of bariatric surgery patients resemble those of lean individuals^58^, which is in agreement with our observations of higher SIgA levels in lean mice and in a majority of patients 1-month post-bariatric surgery.

Overall, we have demonstrated a critical role for IgA in regulating intestinal homeostasis, metabolic inflammation, and obesity-related IR. This work further emphasizes the importance of the intestinal adaptive immune system and its interactions with the gut microbiota and innate immune system within the larger network of organs involved in the manifestation of metabolic disease. Future investigation is required to determine the impact of IgA deficiency during obesity in humans and the role of metabolic disease in human populations with selective IgA deficiency, especially since human IgA deficiency is associated with an altered gut microbiota that cannot be fully compensated with IgM^59^. Nonetheless, our work identifies IgA as a critical immunological molecule in the intestine that impacts systemic glucose homeostasis, and treatments targeting IgA-producing immune populations and SIgA may have therapeutic potential for metabolic disease.

## Methods

### Mice and diet

WT and IgA^−/−^ mice were generated via in-house littermate breeding of C57BL/6J (Jax 664) mice purchased from Jackson Laboratory and C57BL/6J IgA^−/−^ mice obtained from our collaborator, Margaret E. Connor (Baylor College of Medicine, USA). C57BL/6 B cell-deficient muMT^−^ or B^null^ mice (Jax 2288) were purchased from Jackson Laboratory. IgA^−/−^ mice and littermates were weaned and separated according to genotype to control for IgA transmission. We obtained GF mice from McMaster University’s GF facility, which were housed and maintained accordingly in the University of Toronto GF facility. Mice were maintained in a pathogen-free, temperature-controlled, and 12 h light and dark cycle environment at the Toronto Medical Discovery Tower animal research facility. We utilized DIO mouse models to examine metabolic disease in the various groups of mice assessed. All mice used in comparative studies were male, age-matched, littermates, and randomly assigned to either HFD (Research Diets, 60 kcal% fat) or NCD (Envigo, 16 kcal% fat) starting at 6 weeks of age. Investigators were not blinded for glucose and metabolic testing or flow cytometry and qPCR analyses, but were blinded for analysis of histological specimens and microscopy. All animal studies were approved under the Animal Use Protocol (2570.20) by the Animal Care Committee at the University Health Network.

### Bariatric surgery human cohort

Human stool samples were collected at 1) baseline prior to pre-operative run-in with the Optifast diet (800 kcal day^–1^ for 1 week/100lbs body weight) and  bariatric surgery and 2) 1-month visits after surgery from 14 patients. Patient characteristics can be found in Supplementary Table 1. Frozen stool samples were obtained from 10 g fresh stool samples mixed with 20 mL sterile saline (500 mg mL^−1^) filtered through a 300-µm filter, and supernatants from 2 mL aliquots were collected and further diluted (500×) for IgA quantification by ELISA. Human studies were performed with study approval by the Research Ethics Board for Human Subjects (#15-8784) at UHN with informed patient consent.

### Fecal IgA quantification

Small and large bowel fecal contents were harvested and frozen from mice. Pellets were homogenized using Lysing Matrix D tubes (MP Biomedicals) with phosphate-buffered saline (PBS) (100 mg mL^−1^). Homogenates were centrifuged and supernatants and further diluted (1000×) for ELISA quantification for antibody levels (Invitrogen). For human studies, please see methods pertaining to bariatric surgery human cohort.

### Serum isolation

Blood was collected from cardiac puncture or saphenous vein of mice and samples were left at room temperature for 30 min to allow for clotting. Samples were centrifuged for 15 min at 10,000 × *g* to isolate serum for downstream applications.

### Metabolic studies

Body weights, fasting glucose, GTTs, and ITTs were measured as previously described^60^. Mice were fasted overnight for GTTs and fasting insulin (Crystal Chem Inc. ELISA). GTTs and ITTs were performed using 1.5 g glucose per kg body mass and 0.75 U human insulin per kg body mass intraperitoneal injection. Blood glucose measurements were made at 0, 15, 30, 45, 60, 90, and 120 min time-points with a glucometer (Bayer). For metabolic cage studies, mice were placed in automated metabolic cages (Oxymax Systems, Columbus Instruments) for 48 h with constant airflow at 0.5 L per min. Metabolic activity was assessed via indirect calorimetry by recording maximal O_2_ consumption (VO_2_), CO_2_ production (VCO_2_), and heat production (normalized to body weight). Respiratory exchange ratio was calculated as VCO_2_/VO_2_. Ambulatory activity was assessed via the breaking of infrared laser beams in the *x*–*y* plane. Data shown are calculated via the average of light and dark measurements over a 24 h period.

### B cell purification and adoptive transfer

Pan B cells (includes plasma cells) from the spleens, Peyer’s patches, MLN, and small and large intestinal LP of 14–16 week HFD-fed WT and IgA^−/−^ mice were purified using negative selection (>90% purity, EasySep; StemCell Technologies) and injected intraperitoneally (~5.5 × 10^6^ cells) in 12-week HFD-fed muMt^−^ (B^null^) mice.

### Immune cell isolation from tissues

To extract the stromal vascular cell fraction of the VAT, epididymal VAT pads were dissected, collected in 2% fetal bovine serum (FBS) Dulbecco’s modified Eagle’s medium, homogenized in a GentleMACS tissue dissociator (Miltenyi Biotech), and digested in collagenase (0.2 mg mL^−1^, Sigma) at 37 °C, manual shaking every 15 min for a total of 60 min. Cells were the pelleted, washed, filtered through a 70-μm filter, and followed by red blood cell lysis and washing to obtain SVCs.

Intestine LP immune cells were isolated by adapting a protocol previously described^61^. Small (~10 cm prior to the cecum for jejunum and ileum) and large intestines were extracted, removing mesentery fat and Peyer’s patches, and collected in ice-cold wash buffer ((HBSS (Gibco) supplemented with 2% FBS (PAA) and 15 mM HEPES pH 7.4). Extracted intestines were cut open longitudinally into 5 mm pieces in the wash buffer. Bowel pieces were transferred to an EDTA-containing stripping buffer (Hank’s balanced salt solution (HBSS) (Gibco) supplemented with 10% FBS (PAA), 5 mM EDTA, 15 mM HEPES, pH 7.4) and shaken vigorously at 37 °C for 20 min, and then vortexed gently for a few seconds. Supernatant from this step can be collected and passed through a 70-µm nylon cell strainer to obtain the epithelial cell fraction. This wash step was repeated two times in total. Gut pieces were then washed in cold HBBS buffer (Gibco) to remove residual EDTA before transfer into a digestion buffer (RPMI-1640 supplemented with 10% FBS (PAA), 10 mM sodium pyruvate, penicillin–streptomycin antibiotics, collagenase type IV (5 mg mL^−1^, Sigma), and DNase I (0.5 mg mL^−1^, Roche), where it was minced finely with scissors, followed by a 1 h incubation at 37 °C with shaking. The resulting suspension of LP immune cells collected from the previous step were filtered twice through a 100- and 40-µm nylon cell strainer to obtain a single-cell suspension, stimulated with phorbol myristate acetate + (PMA+) stimulation cocktail (Ebioscience) for 5 h (if needed) and/or utilized for downstream applications including flow cytometry and reverse transcription-PCR. Peyer’s patches were excised and four to five patches were combined per sample. For MLN extraction, the lymph nodes adjacent to the colon were extracted. Lymph nodes and Peyer’s patches were mechanically disrupted in PBS and the homogenate was filtered through a 70 µm cell strainer to obtain a single-cell suspension for downstream applications.

### Histology and immunohistochemistry

Mouse VAT, liver, distal small bowel, and colons were fixed for 48 h in 10% buffered formalin prior to processing and hematoxylin and eosin (H&E) staining or immunohistochemistry with a rabbit anti-mouse IgA primary antibody (NSJ Bioreagents R20169) (1:50 dilution in Dako diluent (Dako, Cat. No. S0809)), secondary antibody biotinylated anti-rabbit Ig (Vector Labs, Cat. No. BA-1000), and detection with avidin biotin complex (ABC) system (Vector Labs, Cat. No. PK-6100) and DAB (Abcam, ab64238, DAB Substrate Kit) solution. Fat cell diameter and crown-like structures, defined as adipocytes encircled by several immune cells on H&E staining, were counted with light microscopy or measured using a Leica DFC320 camera with the Leica application suite (LAS) software. Histological images for adipose tissue are shown at ×100 low power field and IgA staining in the small bowel and colon is shown at ×400 high power field and counted by two blinded observers (D.A.W. and S.W.).

### Gene expression assays

Total RNA from cells and tissues was isolated using the RNAeasy Mini Kit (Qiagen) and RT was performed using random primers with the SensiFAST cDNA Synthesis Kit (Bioline). Taqman qPCR gene expression assays were custom designed from Invitrogen and performed according to the manufacturer’s instructions. IDs include Mm03809849_s1, Mm00446345_m1, Mm00657317_m1, Mm00501306_m1, Mm01178820_m1, Mm00446190_m1, Mm01288386_m1, Mm00517640_m1, Mm00505403_m1, Mm00440502_m1, Mm01157588_m1, Mm00440228_gH, and Mm99999915_g1. All other qPCRs were performed on a QuantStudio 6 Flex Real-Time PCR System (Thermo Fisher) using SYBR Green Master Mix reagent (Applied Biosystems). Each sample was run in triplicate and normalized to a housekeeping gene, * Gapdh* (glyceraldehyde 3-phosphate dehydrogenase). Changes in gene expression were calculated using the 2^(−ΔΔCT)^ method using mean cycle threshold values and the housekeeping gene *Gapdh*. The expression of *Gadph*  did not change between groups and results are shown as fold changes relative to the control group. Primer sets utilized for SYBR qPCR assays are listed in Supplementary Table 2.

### Antibiotics and metformin treatment

Mice were administered antibiotics via drinking water ad libitum and bottles were changed twice per week. Water was autoclaved prior to addition of antibiotic cocktail and water intake was monitored daily for the first week to ensure animals’ health. Thereafter, mice were monitored twice a week for 4 weeks of treatment until the end of the study. The antibiotic cocktail consisted of ampicillin (1 g L^−1^), vancomycin (0.5 g L^−1^), neomycin (1 g L^−1^), and metronidazole (1 g L^−1^) (Sigma). Similarly, metformin (Cayman Chemical) was administered through the drinking water ad libitum at a dose of 300 mg kg^−1^ per day.

### Flow cytometry

Single-cell suspensions were stained for 30 min on ice with fluorophore-conjugated commercial antibodies^62^ to CD45.2 (1:200; Cat#109822, clone: 104), CD3 (1:100; Cat#100209, clone: 17A2), CD4 (1:100; Cat#100422, clone: GK1.5), CD8 (1:100; Cat#100710, clone: 53-6.7), γδTcR (1:50; Cat#118118, clone: GL3), Foxp3 (1:50; Cat#320012, clone 150D), IL-17 (1:100; Cat#506903, clone TC11-18H10.1), IFNγ (1:50; Cat#505830, clone: XMG1.2), CD11b (1:125; Cat#101208, clone: M1/70), F4/80 (1:100; Cat#123120, clone: BM8), CD11c (1:125; Cat#117348, clone: N418), CD19 (1:100; Cat#115512, clone: 6D5), B220 (1:100; Cat#103258, clone: RA3-6B2), IgD (1:200; Cat#405714, clone: 11-26c.2a), IgM (1:50; Cat#406531, clone: RMM-1), IgG (1:100; Cat#406001, clone: Poly4060), IgA (1:50; Cat#12-4204-82; Thermo Fisher Scientific eBioscience, clone: mA-6E1), CD206 (1:50; Cat#141710, clone: C068C2), CD80 (1:100; Cat#104708, clone: 16-10A1), CD86 (1:100; Cat#105011, clone: GL-1), CX3CR1 (1:50; Cat#149027, clone: SA011F11), CD103 (1:50; Cat#121416, clone: 2E7), CXCR5 (1:50; Cat#145512, clone L138D7), and I-A^b^ MHC class II (1:125; Cat#116420, clone: AF6-120.1) (BioLegend, unless stated otherwise), followed by viability cell stains with 4′,6-diamidino-2-phenylindole (DAPI) or nuclear stains with Zombie near infrared (NIR) or ultraviolet (UV) (BioLegend). Intracellular staining was performed using a Foxp3 Staining Buffer Kit (eBioscience) for Foxp3, IgA, IgG, IFNγ, and IL-17 markers. Data were collected on a LSRFortessa X-20 flow cytometer (BD Biosciences) and was analyzed with the FlowJo V10 software (Tree Star).

Bacteria fluorescence-activated cell sorting was performed as previously described^55^. Briefly, colonic stool (10–50 mg) was collected fresh from mice and homogenized using Lysis Matrix D tubes (MPBio). Samples were centrifuged at 10,000 × *g* for 1 min at room temperature, and then supernatants were collected and filtered (70 µM). Live bacteria were stained using commercially available SYTO BC Kit (Invitrogen) according to the manufacturer’s instructions at a 1:4000 dilution, followed by bacteria staining with antibody to IgA (eBioscience mA-6E1). For representative gating strategies used in the study, refer to Supplementary Fig. 10.

### Gut permeability assays

FD4 permeability assays were performed by oral gavage of overnight-fasted mice with 0.4 mg g^−1^ body weight of fluorescein isothiocyanate-conjugated dextran (Sigma) and collecting blood in EDTA-coated tubes from the saphenous vein at the 1 and 4 h time-points. Blood plasma was separated by centrifuging the samples at 10,000 × *g* at 4 °C for 15 min. Standards were prepared using plasma spiked with FD4 at varying concentrations. Samples were diluted with PBS 1:1 before loading in a 96-well flat bottom plate and fluorescence was measured at 485/525 nm as previously described^63^.

### Endotoxin measurements

Serum endotoxin levels were measured using a Pyrogene Recombinant C Endotoxin Detection Fluorescence Kit (Lonza; Cat# 50-658U) as per the manufacturer’s instructions.

### Microbial encroachment studies

Using the methanol-Carnoy’s fixation method, colons containing fecal pellets were fixed and processed for FISH and immunostaining. Briefly, colons containing fecal pellets were extracted in methanol-Carnoy’s fixative (60% (v/v) dry methanol, 30% (v/v) chloroform, 10% (v/v) glacial acetic acid) for up to 2 weeks. Tissues were washed twice in dry methanol (30 min), twice in absolute ethanol (20 min), and incubated in xylene bath for 15 min prior to paraffin embedding and sectioning (4 µm thick) at the STARR histology core facility. For FISH, sectioned slides were dewaxed by incubating in 60 °C for 10 min, followed by incubation with prewarmed xylene substitute solution for 10 min twice. Slides were then incubated in 99.5% ethanol for 5 min and let to air dry. The bacteria EUB338 probe (5′-GCTGCCTCCCGTAGGAGT-3′) and a nonsense probe as control (5′-CGACGGAGGGCATCCTCA-3′) (Alexa647, Thermo Fisher) (0.5 µg) was mixed with 50 µL of prewarmed hybridization solution (20 mM Tris-HCl, pH 7.4, 0.9 M NaCl, 0.1% (w/v) SDS, 5% (v/v) formamide), heated to 50 °C, and then added as drops to the dried slides. Slides were covered with coverslips and placed in a humid chamber at 50 °C overnight. Coverslips were carefully removed and slides were incubated in the FISH washing buffer (20 mM Tris-HCl, pH 7.4, 0.9 M NaCl) twice for 15 min at 50 °C, followed by three washes with PBS for 10 min each. Immunofluoresence staining was then performed. Samples were marked with a PAP pen on the slides and block solution (5% FBS in PBS) was added to the slides and incubated in the dark in a humid chamber for 30 min at 4 °C. The MUC2 (anti-MUC2 antibody (Novus Biologicals)) was diluted 1:1500 with a block solution and added to the slide. Slides were incubated overnight at 4 °C and then washed with PBS three times for 10 min each. The anti-rabbit Alexa488 secondary antibody (Cat# A-11008, Invitrogen) was diluted 1:500 in block solution, added to the sample, and then incubated for 2 h in 4 °C. Slides were washed three times with PBS for 10 min each prior to mounting with mounting media (ProLong Diamond Antifade with DAPI, Thermo Fisher) and set at room temperature. Slides were analyzed using the AxioImager (Zeiss), and ImageJ software was used to quantify distance of bacteria from intestinal epithelium taking ten measurements per image and averaged per sample.

### 16S bacterial load assay

DNA was isolated from mouse fecal pellets by using the Qiagen DNeasy PowerSoil Kit (Qiagen), according to the manufacturer’s instructions. DNA was eluted in 35 μL of provided elution buffer. Densities were measured using a 16S qPCR^64^. Amplification and detection by real-time PCR were performed with the QuantStudio 6 Flex Real-Time PCR System (Thermo Fisher). Duplicate samples were used for determination of DNA by real-time PCR. All samples were analyzed in duplicate in a 10 μL reaction volume using the TaqMan Universal PCR Master Mix (Applied Biosystems), containing 300 nM of each of the universal forward and reverse primers and 200 nM of the fluorogenic probe. The reaction conditions for amplification of DNA were 95 °C for 10 min, and 45 cycles of 95 °C for 15 s and 60 °C for 1 min.

Data analysis was done with the QuantStudio Real-Time PCR Software version 1.3 (Applied Biosystems). Cycle threshold (Ct) values were reported as a representative estimation of bacterial load^64^. Lower limit of detection was determined as Ct from negative DNA extraction protocol control Primer and probe sequences are listed in Supplementary Table 2.

### Fecal transplantation studies

For fecal transplantation studies, antibiotics were given as previously described^65^. Briefly, a systemic cocktail consisting of ampicillin (Cayman Chemical), cefoperazone (UHN Pharmacy) and clindamycin (UHN Pharmacy), and a non-absorbing cocktail consisting of vancomycin (Cayman Chemical), ertapenem (Invanz, Merck), and neomycin (Cayman Chemicals) were given in alternating weeks in the following sequence detailed below. On week 1, WT mice fed HFD for 8 weeks received the systemic cocktail, switching to non-absorbing on week 2, followed by the systemic cocktail again on week 3 with 2 days in between each week to receive only regular drinking water. All antibiotics were administered at 1 mg mL^−1^ in drinking water. Following 2 days after the complete 3-week antibiotic conditioning regimen, mice received a single 200 µL oral gavage with colonic fecal matter (diluted 1:5 in 10% glycerol saline) from either IgA WT or IgA^−/−^ mice fed a HFD for 14–16 weeks. Four weeks after the gavage, mice were subject to metabolic testing. Nine-week-old GF mice fed a HFD for 2 weeks were singly housed in the University of Toronto GF facility and received the same fecal matter transfer and metabolic testing as described above.

### 16S rRNA gene sequencing

Bacterial DNA was isolated from stool samples using the DNA Stool Mini Kit (Qiagen). The V4 hypervariable region of the 16S rRNA gene is amplified using a universal forward sequencing primer and a uniquely barcoded reverse sequencing primer to allow for multiplexing^66^. Amplification reactions are performed using 12.5 µL of KAPA2G Robust HotStart ReadyMix (KAPA Biosystems), 1.5 µL of 10 µM forward and reverse primers, 8.5 µL of sterile water, and 2 µL of DNA. The V4 region was amplified by cycling the reaction at 95 °C for 3 min, 24× cycles of 95 °C for 15 s, 50 °C for 15 s, and 72 °C for 15 s, followed by a 5 min 72 °C extension. All amplification reactions were done in triplicate, checked on a 1% agarose TBE gel, and then pooled to reduce amplification bias. Pooled triplicates were quantified using Quant-it PicoGreen dsDNA Assay (Thermo Fisher Scientific) and combined by even concentrations. The final library was purified using Ampure XP beads (Agencourt), selecting for the bacterial V4 amplified band. The purified library was quantified using Qubit dsDNA assay (Thermo Fisher Scientific) and loaded onto the Illumina MiSeq for sequencing, according to the manufacturer’s instructions (Illumina, San Diego, CA). Sequencing was performed using the V2 (150 bp × 2) chemistry.

### Analysis of the bacterial microbiome

The UNOISE pipeline, available through USEARCH version 9.2, was used for sequence analysis^67^. The last base, typically error prone, was removed from all the sequences. Sequences were assembled and quality trimmed using –fastq_mergepairs and –fastq_filter, with a –fastq_maxee set at 1.0 and 0.5, respectively. Assembled sequences <233 bp were removed. Following the UNOISE pipeline, merged pairs were then de-replicated and sorted to remove singletons using the vsearch software^68^. Sequences were denoised and chimeras were removed using the unoise2 command in USEARCH v. 9.2. Assembled sequences were then mapped back to the chimera-free denoised sequences at 97% identity Operational Taxonomic Units (OTUs), using vsearch. Taxonomy assignment was executed using utax, available through USEARCH, and the UNOISE compatible Ribosomal Database Project (RDP) database version 16, with a minimum confidence cut-off of 0.9^69^. OTU sequences were aligned using PyNast accessed through QIIME^70^. Sequences that did not align were removed from the dataset and a phylogenetic tree of the filtered aligned sequence data was made using FastTree^71^. Low abundance OTUs (<0.005% RA) were removed from the OTU table^72^.

α-Diversity and rarefactions were calculated using QIIME^70^. PCA plots were of the rarefied data using QIIME and plotted using EMPeror^73^. The OTUs were summarized at the genus level and input into the LEfSe program to determine biomarkers associated with groups of interest^74^.

### Statistics

Dataset normality was confirmed by Shapiro–Wilk normality test. For data that passed normality testing, statistical difference between two means was determined via two-sided, unpaired *t* test, otherwise a Mann–Whitney test was used (GraphPad Prism Software Inc.). In figure legends, the *n* value specified indicates the number of mice, unless stated otherwise. Figures displaying pooled results of more than one experiment are explicitly stated for each figure. Sample size was not predetermined for either mouse or human studies, but we performed experiments with group sizes based on existing published literature of similar experiments. An extreme studentized deviate method (Grubbs’ test) was performed to assess for statistical outliers. All data are presented as means ± SEM. Statistical significance was set at *p* < 0.05. * denotes *p* < 0.05, ** denotes *p* < 0.01, and *** denotes *p* < 0.001.

### Reporting summary

Further information on research design is available in the Nature Research Reporting Summary linked to this article.

## Supplementary information

Supplementary Information

Reporting Summary

Source Data

## Data Availability

16S rRNA sequence data are deposited in BioProject under the accession code PRJNA548206. All other data generated or analyzed during this study are included in this published article and its Supplementary information files or are available from the corresponding authors upon reasonable request.
