# Supplementary material for: Gut-associated IgA+ immune cells regulate obesity-related insulin resistance
Source: Nat Commun. 2019 Aug 13;10:3650. doi: 10.1038/s41467-019-11370-y (PMC6692361; doi:10.1038/s41467-019-11370-y)
Supplement: Supplementary file 1 — Supplementary Information [file 41467_2019_11370_MOESM1_ESM.pdf]

“Gut associated IgA immune populations regulate obesity related insulin resistance”

Luck and Khan et al.

Supplementary Information

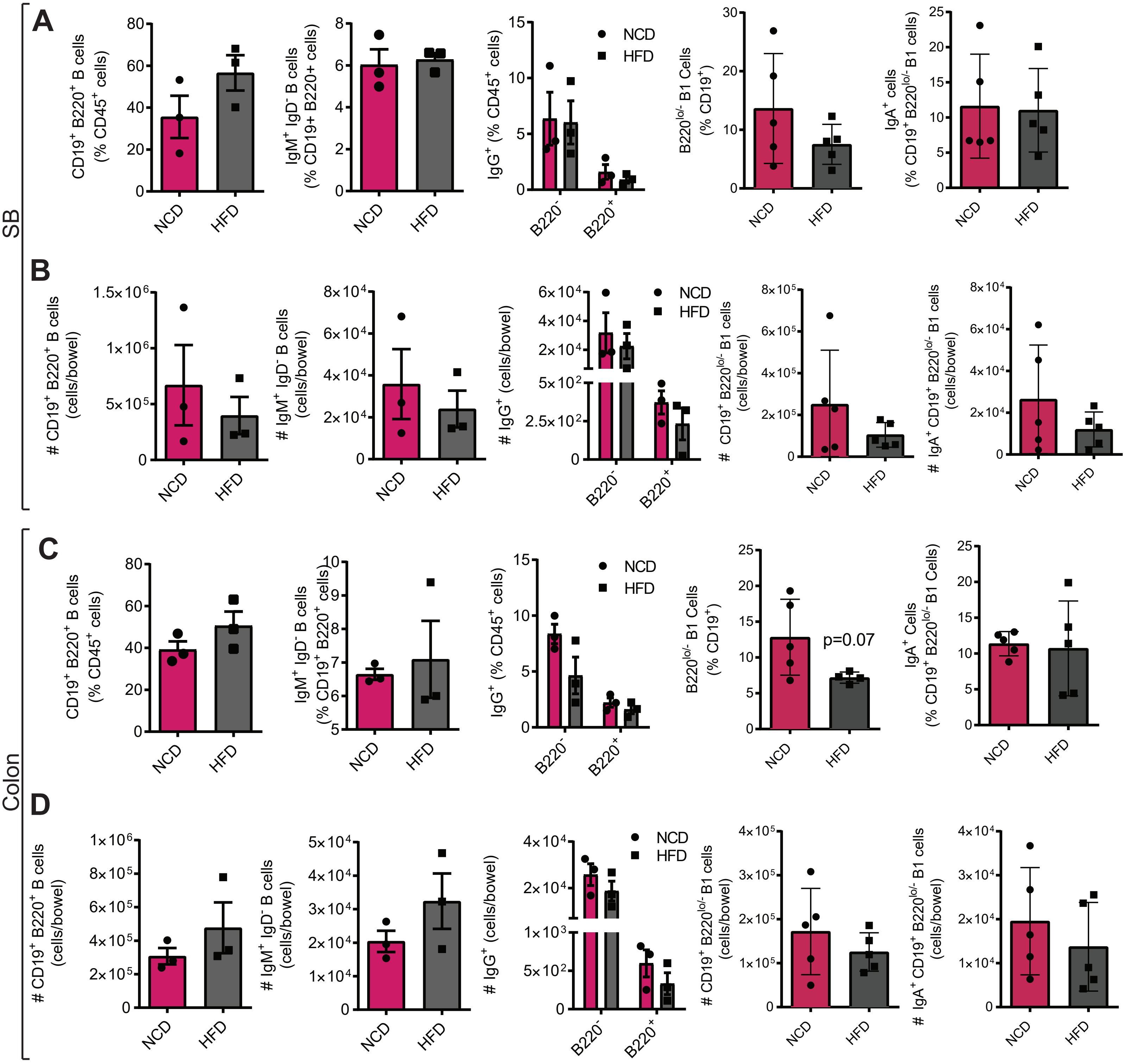

Supplementary Figure 1. B cell populations within the small and large bowel of HFD-fed mice. A) Frequency and **B)** absolute numbers of CD19⁺ B220⁺ total B cells (far left), IgM⁺ IgD⁻ B cells (middle left), IgG⁺ B220⁺ B cells, IgG⁺ B220⁻ plasma cells (middle), CD19⁺ B220$^{lo/-}$ B1 cells (middle right), and IgA+ CD19⁺ B220$^{lo/-}$ B1 cells (far right) in the distal small intestine LP of HFD-fed mice compared to NCD controls (n=3-5/group). **C)** Frequency and **D)** absolute numbers of CD19⁺ B220⁺ total B cells (far left), IgM⁺ IgD⁻ B cells (middle left), IgG⁺ B220⁺ B cells, IgG⁺ B220⁻ plasma cells (middle), CD19⁺ B220$^{lo/-}$ B1 cells (middle right), and IgA+ CD19⁺ B220$^{lo/-}$ B1 cells (far right) in the colon LP of HFD-fed mice compared to NCD controls (n=3-5/group). Data are means ± SEM.

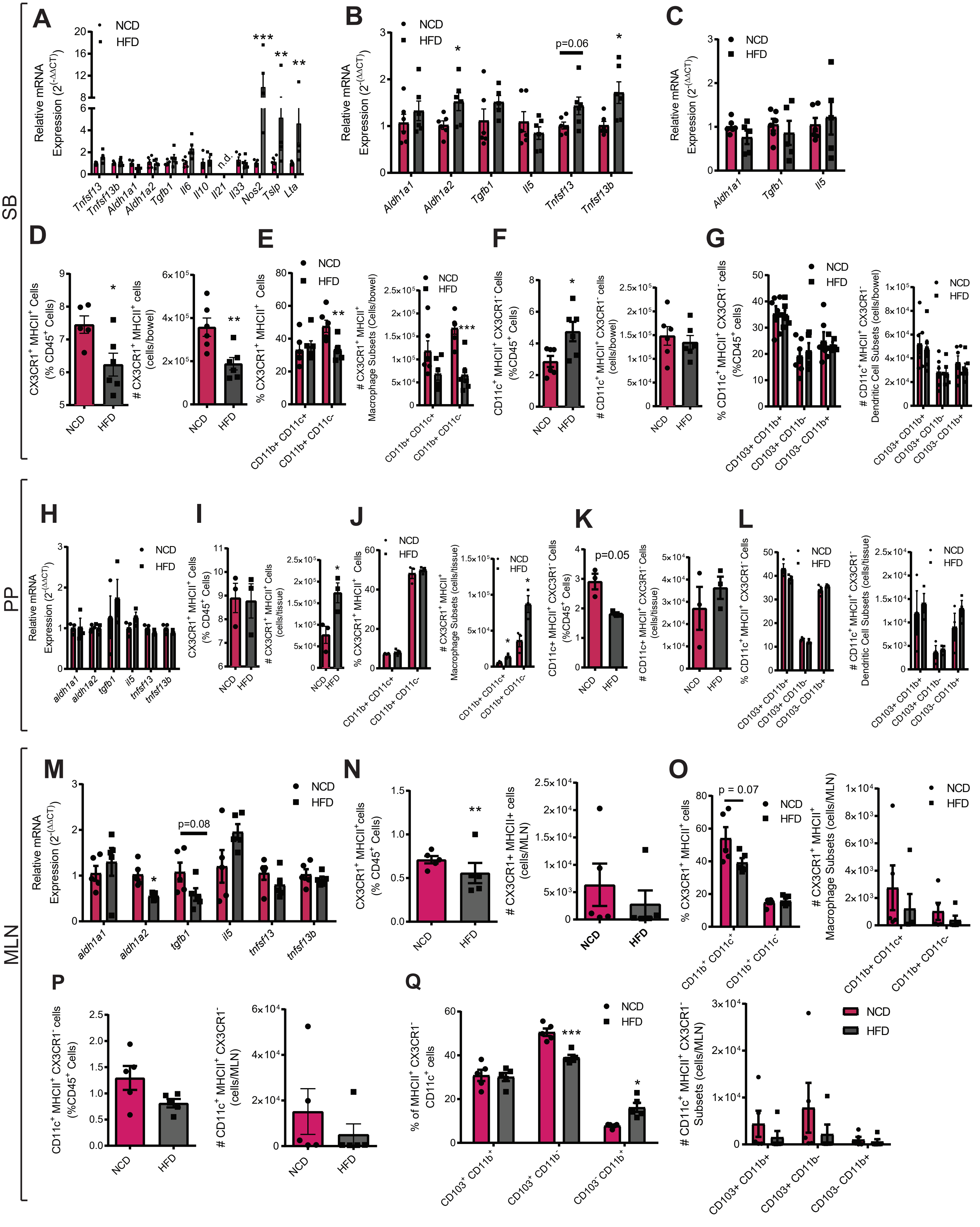

SB
A
NCD
HFD
Relative mRNA Expression (2^-(ΔΔCT))
***
**
**
n.d.
Tnfsf13
Tnfsf13b
Aldh1a1
Aldh1a2
Tgfb1
Il6
Il10
Il21
Il33
Nos2
Tslp
Lta
B
NCD
HFD
Relative mRNA Expression (2^-(ΔΔCT))
p=0.06
Aldh1a1
Aldh1a2
Tgfb1
Il5
Tnfsf13
Tnfsf13b
C
NCD
HFD
Relative mRNA Expression (2^-(ΔΔCT))
Aldh1a1
Tgfb1
Il5
D
CX3CR1+ MHCII+ Cells (% CD45+ Cells)
CX3CR1+ MHCII+ cells (cells/bowel)
NCD
HFD
E
NCD
HFD
% CX3CR1+ MHCII+ Cells
# CX3CR1+ MHCII+ Macrophage Subsets (Cells/bowel)
CD11b+ CD11c+
CD11b+ CD11c-
F
CD11c+ MHCII+ CX3CR1- Cells (%CD45+ Cells)
CD11c+ MHCII+ CX3CR1- cells (cells/bowel)
G
NCD
HFD
% CD11c+ MHCII+ CX3CR1- cells (%CD45+ cells)
# CD11c+ MHCII+ CX3CR1- Dendritic Cell Subsets (cells/bowel)
CD103+ CD11b+
CD103+ CD11b-
CD103- CD11b+
PP
H
NCD
HFD
Relative mRNA Expression (2^-(ΔΔCT))
aldh1a1
aldh1a2
tgfb1
il5
tnfsf13
tnfsf13b
I
CX3CR1+ MHCII+ Cells (% CD45+ Cells)
CX3CR1+ MHCII+ Cells (cells/tissue)
J
% CX3CR1+ MHCII+ Cells
# CX3CR1+ MHCII+ Macrophage Subsets (cells/tissue)
K
p=0.05
CD11c+ MHCII+ CX3CR1- Cells (%CD45+ Cells)
CD11c+ MHCII+ CX3CR1- Cells (cells/tissue)
L
% CD11c+ MHCII+ CX3CR1- Cells
# CD11c+ MHCII+ CX3CR1- Dendritic Cell Subsets (cells/tissue)
MLN
M
Relative mRNA Expression (2^-(ΔΔCT))
p=0.08
N
CX3CR1+ MHCII+cells (% CD45+ Cells)
CX3CR1+ MHCII+ cells (cells/MLN)
O
p = 0.07
% CX3CR1+ MHCII+ cells
# CX3CR1+ MHCII+ Macrophage Subsets (cells/MLN)
CD11b+ CD11c+
CD11b+ CD11c-
P
CD11c+ MHCII+ CX3CR1- cells (%CD45+ Cells)
CD11c+ MHCII+ CX3CR1- (cells/MLN)
Q
% of MHCII+ CX3CR1- CD11c+ cells
# CD11c+ MHCII+ CX3CR1- Subsets (cells/MLN)
CD103+ CD11b+
CD103+ CD11b-
CD103- CD11b+
NCD
HFD

Supplementary Figure 2. IgA promoting mediators the small intestine, PP and MLN of HFD-fed mice. Relative mRNA expression of genes promoting IgA in small intestine **A)** whole tissue (n=5/group, 2 experiments), **B)** lamina propria (LP) (n=6/group, 2 experiments) and **C)** epithelial cell fraction (n=6 NCD, 5 HFD, 2 experiments) in HFD-fed C57BL/6J mice after 14 weeks compared to NCD controls. Frequency (left) and absolute number (right) of **D)** $CX3CR1^{+}$ $MHCII^{+}$ macrophages, **E)** corresponding macrophage subsets, **F)** $CD11c^{+}$ $MHCII^{+}$ $CX3CR1^{-}$ dendritic cells and **G)** corresponding dendritic cell subsets in the small intestinal LP of C57BL/6J mice fed a HFD/NCD for 14 weeks (n=6, 2 experiments). **H)** Relative mRNA expression of genes promoting IgA in Peyer's patches of WT HFD/NCD fed mice (n=3/group). Frequency (left) and absolute number (right) of **I)** $CX3CR1^{+}$ $MHCII^{+}$ macrophages, **J)** corresponding macrophage subsets**, K)** $CD11c^{+}$ $MHCII^{+}$ $CX3CR1^{-}$ dendritic cells and **L)** corresponding dendritic cell subsets in the Peyer's patches of WT mice fed a HFD for 14 weeks compared to NCD controls (n=3/group). **M)** Relative mRNA expression of genes promoting IgA in colon draining MLN of WT HFD/NCD fed mice (n=5/group). Frequency (left) and absolute number (right) of **N)** $CX3CR1^{+}$ $MHCII^{+}$ macrophages, **O)** corresponding macrophage subsets**, P)** $CD11c^{+}$ $MHCII^{+}$ $CX3CR1^{-}$ dendritic cells and **Q)** corresponding dendritic cell subsets in the MLN of WT mice fed a HFD for 14 weeks compared to NCD controls (n=5/group, 2 experiments). Data are means ± SEM. * denotes $p<0.05$, ** denotes $p<0.01$ and *** denotes $p<0.001$.

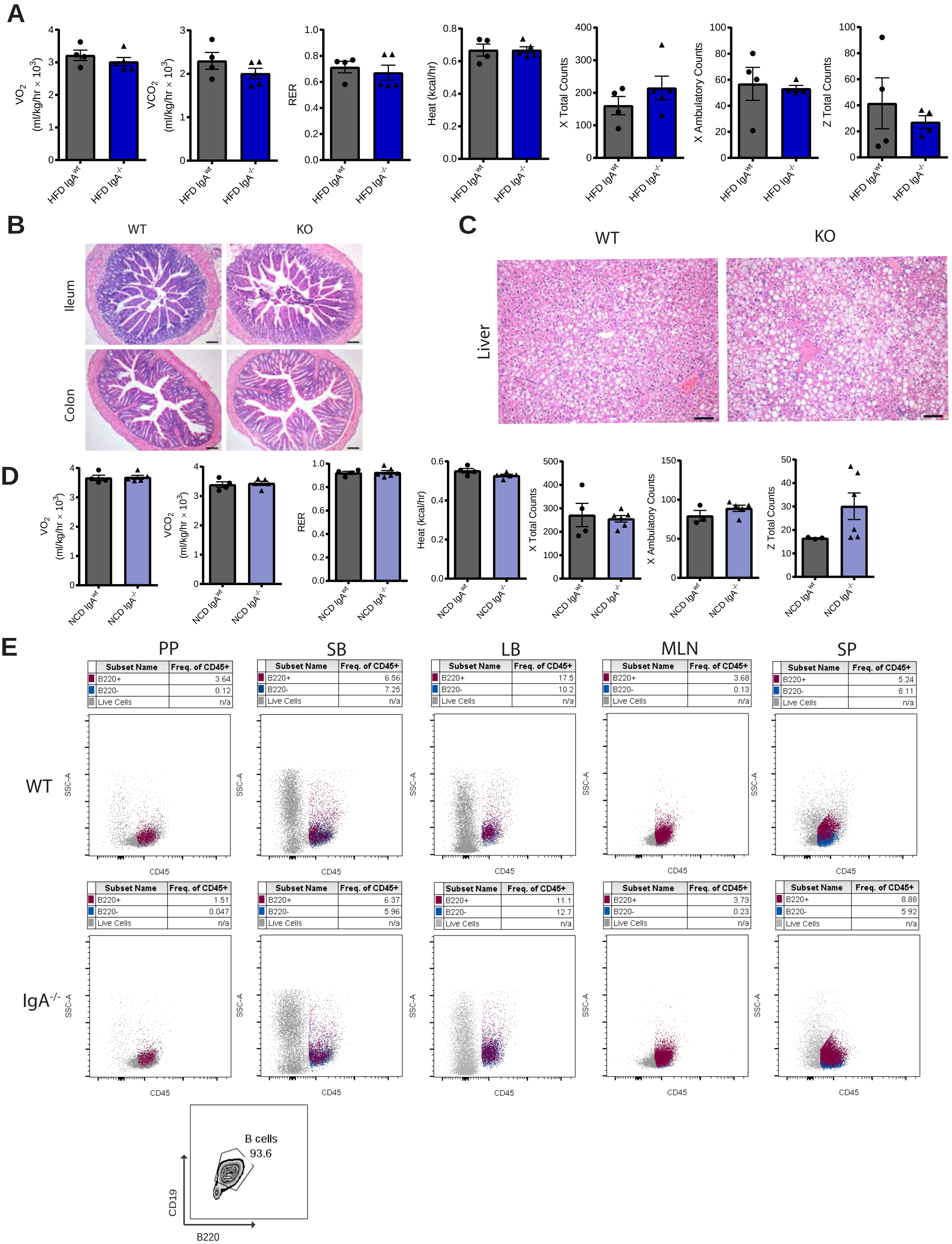
A
VO2 (ml/kg/hr × 10^3)
VCO2 (ml/kg/hr × 10^3)
RER
Heat (kcal/hr)
X Total Counts
X Ambulatory Counts
Z Total Counts
HFD IgA^wt
HFD IgA^-/-
B
WT
KO
Ileum
Colon
C
WT
KO
Liver
D
VO2 (ml/kg/hr × 10^3)
VCO2 (ml/kg/hr × 10^3)
RER
Heat (kcal/hr)
X Total Counts
X Ambulatory Counts
Z Total Counts
NCD IgA^wt
NCD IgA^-/-
E
PP
SB
LB
MLN
SP
WT
IgA^-/-
Subset Name
Freq. of CD45+
B220+
B220-
Live Cells
n/a
WT: PP B220+ 3.64, B220- 0.12; SB B220+ 6.56, B220- 7.25; LB B220+ 17.5, B220- 10.2; MLN B220+ 3.68, B220- 0.13; SP B220+ 5.24, B220- 8.11
IgA^-/-: PP B220+ 1.51, B220- 0.047; SB B220+ 6.37, B220- 5.96; LB B220+ 11.1, B220- 12.7; MLN B220+ 3.73, B220- 0.23; SP B220+ 8.88, B220- 5.92
SSC-A
CD45
B cells
93.6
CD19
B220

Supplementary Figure 3. Histological and metabolic assessment of obese IgA$^{-/-}$ mice and reconstitution and purity assessment of adoptive transfer studies. A) Metabolic parameters including (from left to right) O_2 consumption, CO_2 output, respiratory exchange ratio (RER), heat production, and X total, X ambulatory and Z total movement activity in 14 week HFD-fed vs. WT controls (n=4 WT, n=5 IgA$^{-/-}$ HFD mice, 2 experiments). **B)** Representative histological images of the ileum (top) and colon (bottom) of WT (left) vs. IgA$^{-/-}$ (KO) (right) mice fed HFD for 14 weeks with the scale bar set at 100µm. **C)** Representative histological images of liver sections of WT (left) vs. IgA$^{-/-}$ (KO) (right) mice fed HFD for 14 weeks. **D)** Metabolic parameters of NCD-fed IgA$^{-/-}$ vs WT controls as described in A) (n=4 WT and n=6 IgA$^{-/-}$ NCD-fed mice, 2 experiments) **E)** Representative reconstitution plots in intestinal and associated lymphoid tissues (SB, LB, MLN, PP and Spleen) of µMT$^{-/-}$ (B^{null}) recipients receiving either WT or IgA-/- pan B cells (B220^{+} and B220^{-}) 1 week post-transfer and representative plot of purity (>93%) of B cell enrichment for intestinal B cell adoptive transfers (bottom plot). Data are means ± SEM.

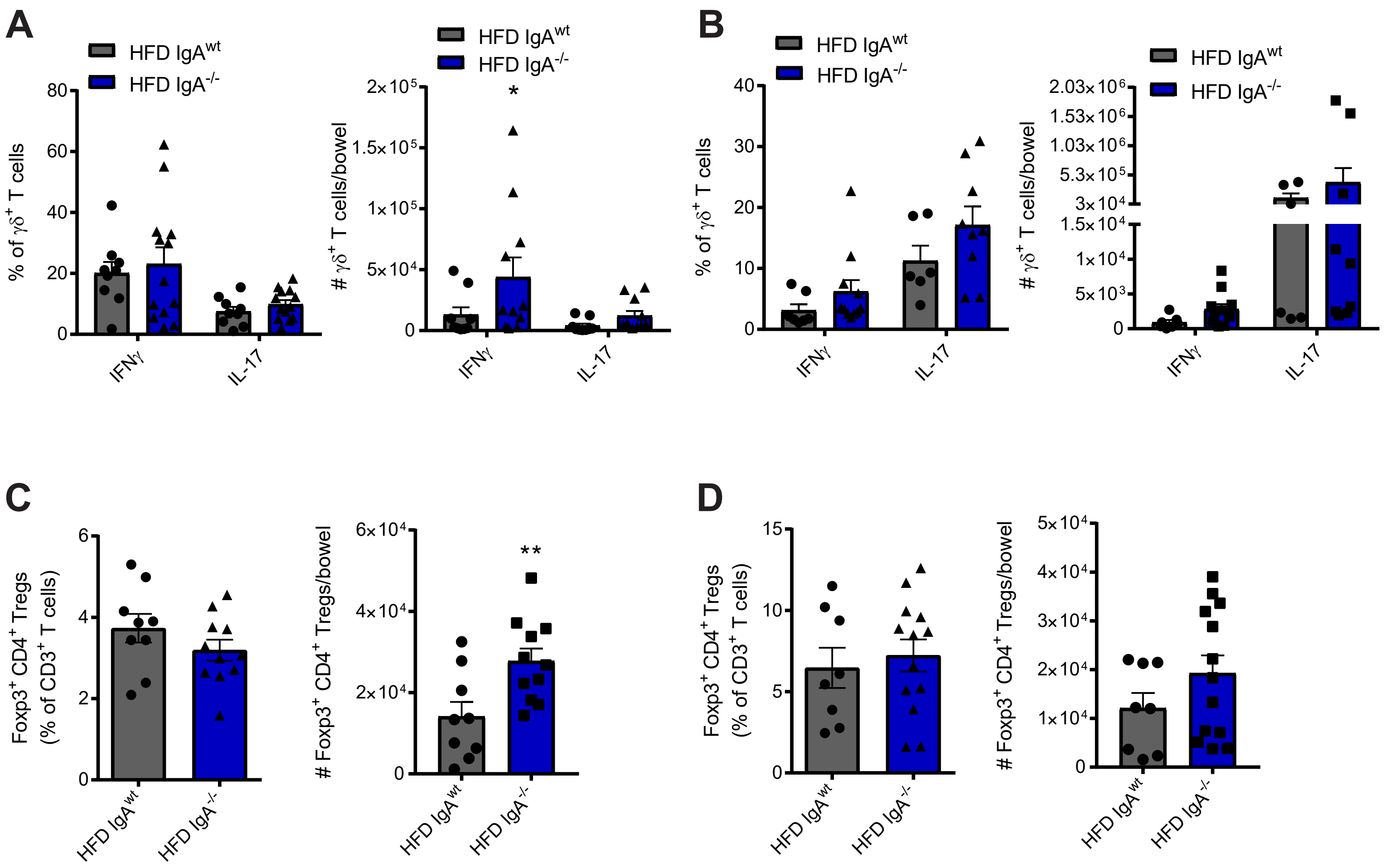

Supplementary Figure 4. Intestinal γδ⁺ T cells and regulatory T cells in HFD-fed IgA$^{-/-}$ mice. Frequency (left) and absolute number (right) of IFNγ and IL-17 producing γδ⁺ T cells in the **A)** distal small intestine and **B)** colon lamina propria (LP) of IgA$^{-/-}$ compared to WT mice after 14 weeks of HFD feeding (n=6-9 WT, 9-11 IgA$^{-/-}$, 3-4 experiments). Frequency (left) and absolute number (right) of Foxp3⁺ CD4⁺ Tregs in the **C)** distal small intestine and **D)** colon LP of IgA$^{-/-}$ compared to WT mice after 14 weeks of HFD feeding (n= 8-9 WT, 11-13 IgA$^{-/-}$, 3-4 experiments)Data are means ± SEM. * denotes $p<0.05$ and ** denotes $p<0.01$.

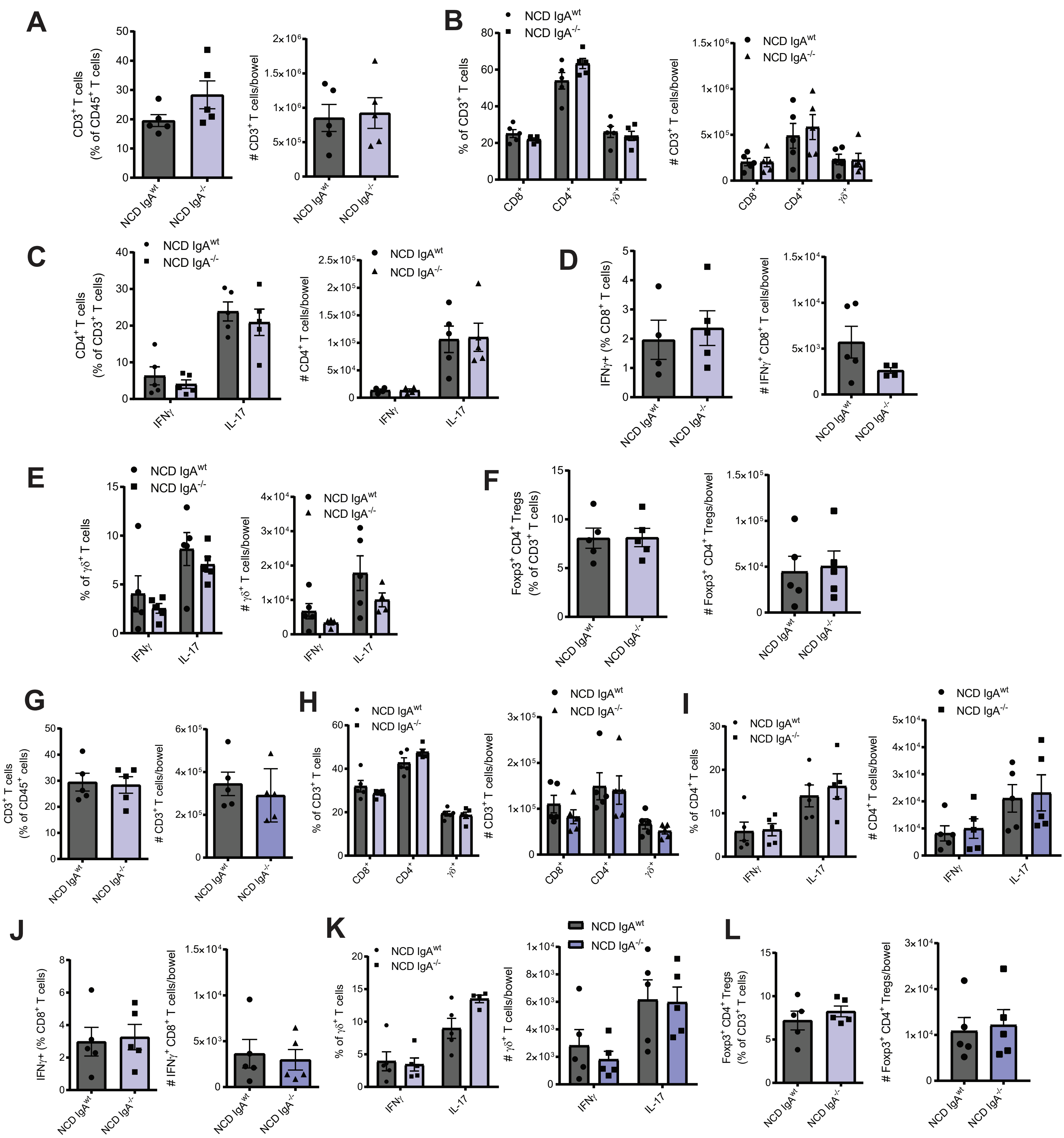

Supplementary Figure 5. Intestinal T cell populations in NCD-fed IgA deficient mice. Frequency (left) and absolute number (right) of **A)** $CD3^+$ T cells, **B)** T cell subsets, **C)** IFNγ and IL-17 producing (Th1 and Th17, respectively) $CD4^+$ T cells, **D)** IFNγ producing $CD8^+$ T cells, **E)** IFNγ and IL-17 producing $\gamma\delta^+$ T cells and **F)** $Foxp3^+$ $CD4^+$ Tregs in the distal small intestinal lamina propria (LP) of HFD-fed $IgA^{-/-}$ compared to WT controls (n=5/group). **G)** Frequency (left) and absolute number (right) of $CD3^+$ T cells, **H)** T cell subsets, **I)** IFNγ and IL-17 producing (Th1 and Th17, respectively) $CD4^+$ T cells, **J)** IFNγ producing $CD8^+$ T cells, **K)** IFNγ and IL-17 producing $\gamma\delta^+$ T cells and **L)** $Foxp3^+$ $CD4^+$ Tregs in colon LP of HFD-fed $IgA^{-/-}$ compared to WT controls (n=5/group). Data are means ± SEM.

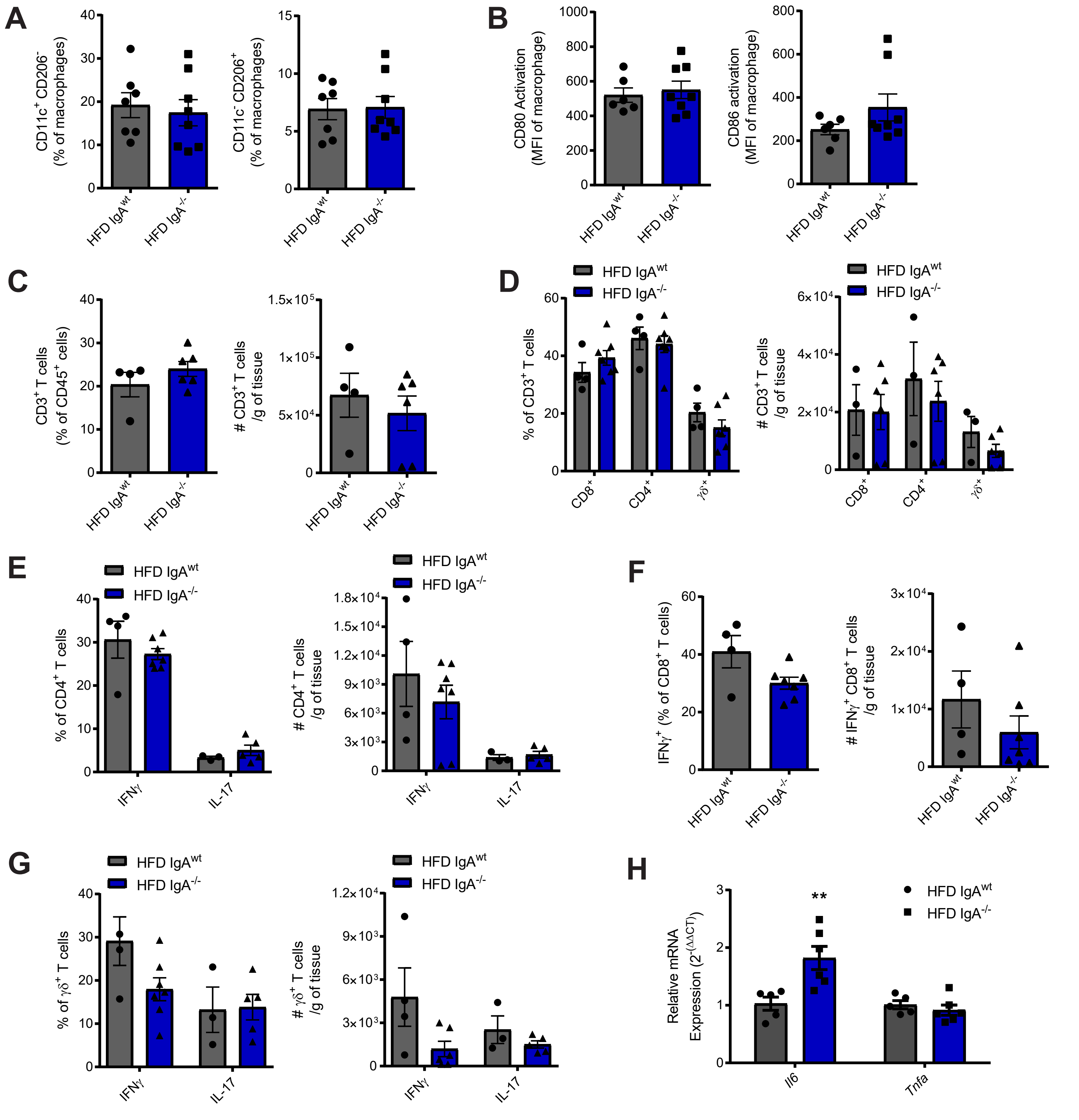

Supplementary Figure 6. VAT macrophage subsets and T cells, and IL-6/TNFα expression in HFD-fed IgA$^{-/-}$ mice. A) Percentages of M1 (left) and M2 (right) macrophage subsets within the visceral adipose tissue (VAT) stromal vascular fraction (n=7 WT, 8 IgA$^{-/-}$ pooled mice, 3 experiments). **B)** Expression of CD80 and CD86 activation in total macrophages shown by MFI (n=6 WT, 8 IgA$^{-/-}$ pooled mice, 3 experiments). Frequency (left) and absolute number (right) of **C)** CD3^{+} T cells, **D)** T cell subsets, **E)** IFNγ and IL-17 producing CD4^{+}, **F)** IFNγ producing CD8^{+}, and **G)** IFNγ and IL-17 producing γδ^{+} T cells in the stromal vascular cells of VAT in IgA$^{-/-}$ mice fed HFD for 14 weeks compared to WT controls (n=3-4 WT, 5-7 IgA$^{-/-}$ pooled mice; 4 experiments). **H)** mRNA expression levels of IL-6 and TNFα within VAT (n=5 WT, 6 IgA$^{-/-}$ mice, 2 experiments). Data are means ± SEM. ** denotes $p<0.01$.

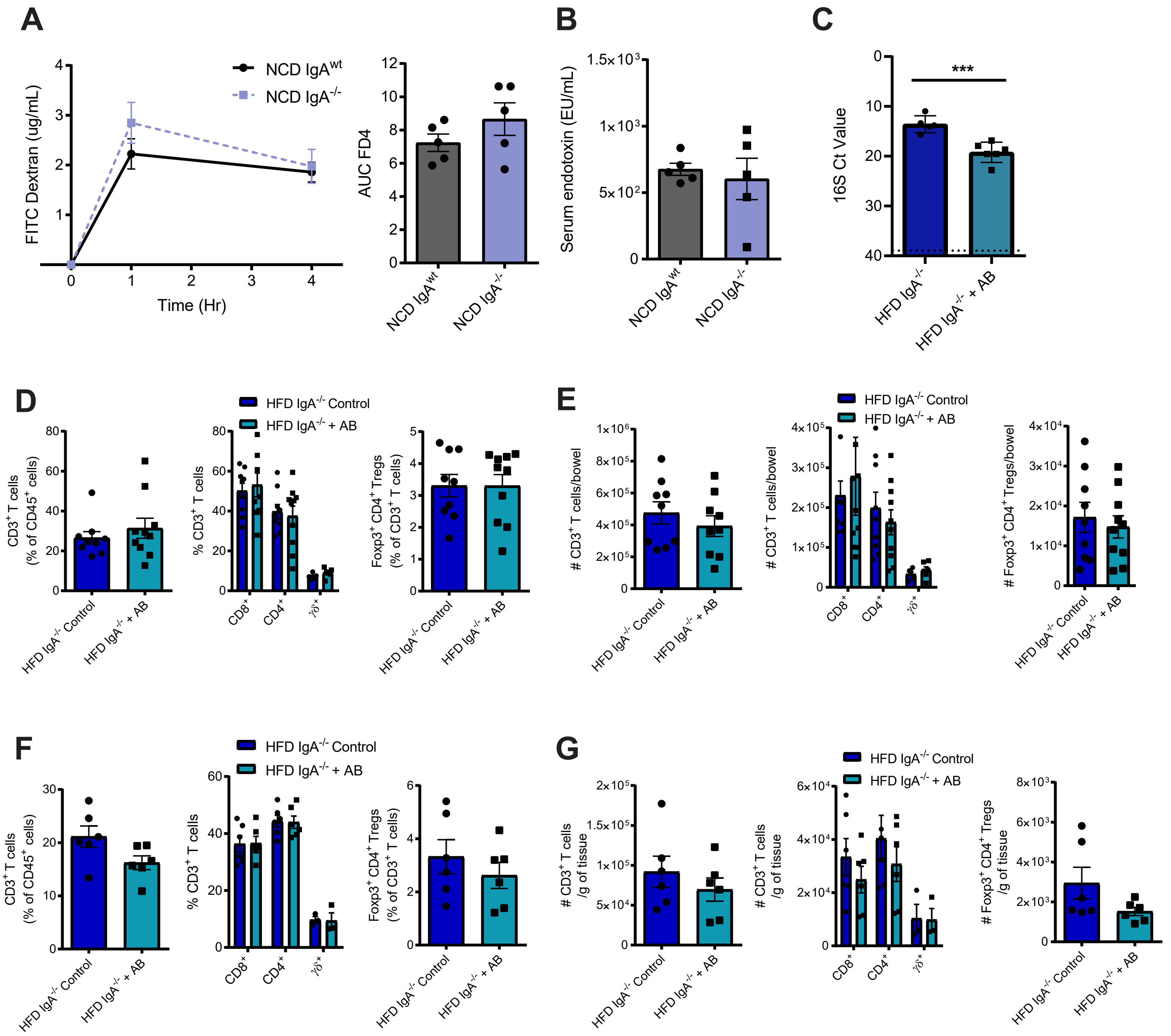

Supplementary Figure 7. Measurement of intestinal barrier function in of NCD-IgA$^{-/-}$ mice, and 16S fecal bacterial density and gut and VAT T cell populations in obese IgA$^{-/-}$ mice treated with antibiotics. A) Intestinal permeability assay measuring concentration of FITC labelled-dextran by fluorescence (left) also represented by area under the curve (AUC) (right) in collected plasma 1 and 4 hours post-gavage in NCD-fed mice (n=5/group) and **B)** serum endotoxin levels in IgA-/- mice treated with antibiotics in drinking water for 4 weeks compared to untreated NCD IgA-/- controls (n=5/group). **C)** Bacterial 16S Ct values from feces of HFD-fed IgA$^{-/-}$ mice treated with antibiotics compared to HFD-fed IgA$^{-/-}$ untreated controls (n=5 WT, 6 IgA$^{-/-}$ mice, 2 experiments). Distal small intestinal **D)** frequency and **E)** absolute number of CD3$^+$ T cells (left), T cell subsets (middle) and Foxp3$^+$ CD4$^+$ regulatory T cells (Tregs) (right) in HFD-fed IgA$^{-/-}$ mice treated with antibiotics in drinking water for 4 weeks compared to HFD-fed IgA$^{-/-}$ untreated controls (n=9 WT, 10 IgA$^{-/-}$ mice, 3 experiments). Visceral adipose tissue **F)** frequency and **G)** absolute number of CD3$^+$ T cells (left), T cell subsets (middle) and Foxp3$^+$ CD4$^+$ regulatory T cells (Tregs) (right) within the stromal vascular fraction of HFD-fed IgA$^{-/-}$ mice treated with antibiotics compared to HFD-fed IgA$^{-/-}$ untreated controls (n=6 pooled mice/group, 2 experiments). Data are means ± SEM. *** denotes p<0.001.

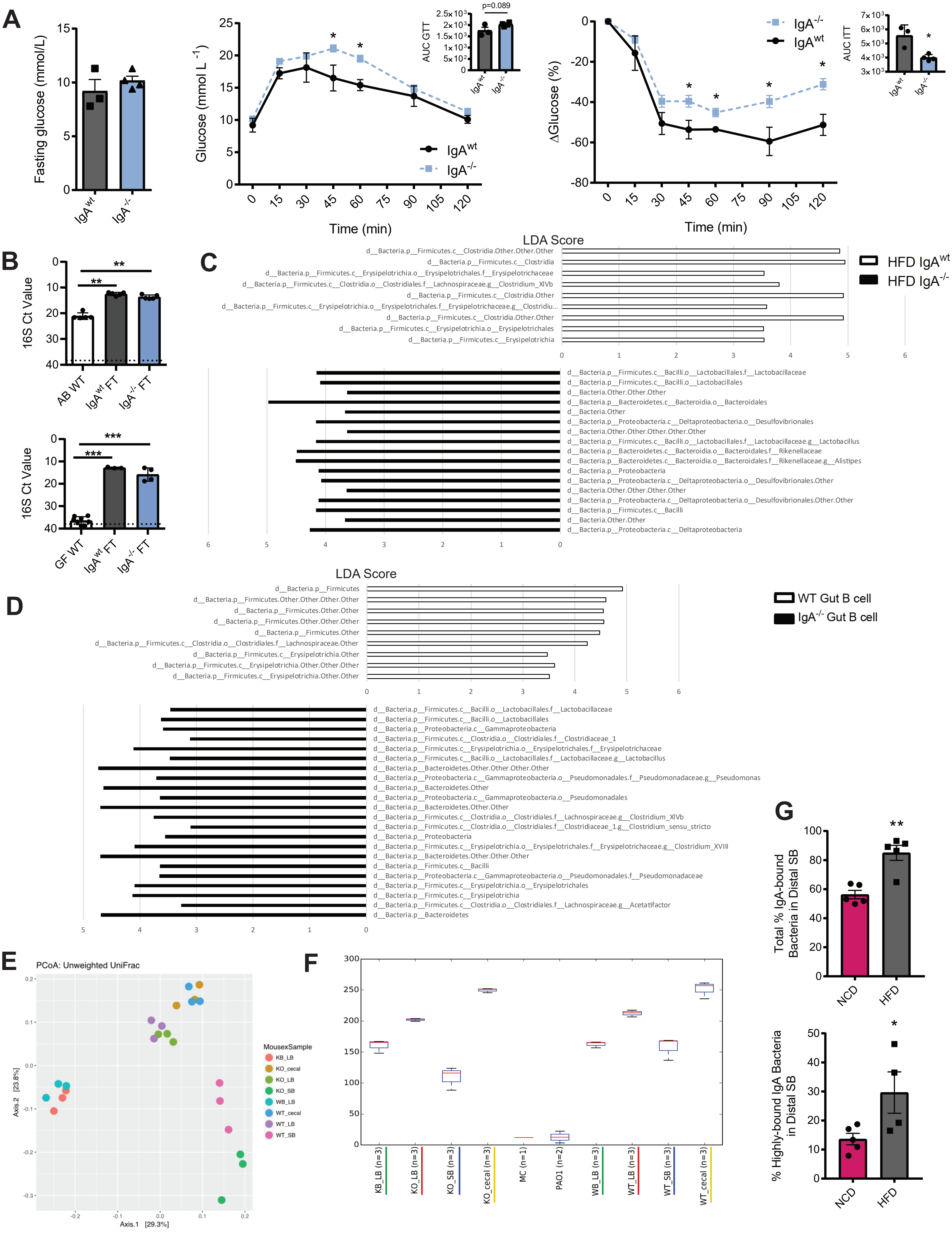
A
Fasting glucose (mmol/L)
IgA wt
IgA -/-
Glucose (mmol L -1)
Time (min)
AUC GTT
p=0.089
IgA wt
IgA -/-
ΔGlucose (%)
AUC ITT
B
16S Ct Value
AB WT
IgA wt FT
IgA -/- FT
GF WT
C
LDA Score
HFD IgA wt
HFD IgA -/-
d__Bacteria.p__Firmicutes.c__Clostridia.Other.Other.Other
d__Bacteria.p__Firmicutes.c__Clostridia
d__Bacteria.p__Firmicutes.c__Erysipelotrichia.o__Erysipelotrichales.f__Erysipelotrichaceae
d__Bacteria.p__Firmicutes.c__Clostridia.o__Clostridiales.f__Lachnospiraceae.g__Clostridium_XlVb
d__Bacteria.p__Firmicutes.c__Clostridia.Other
d__Bacteria.p__Firmicutes.c__Erysipelotrichia.o__Erysipelotrichales.f__Erysipelotrichaceae.g__Clostridiu...
d__Bacteria.p__Firmicutes.c__Clostridia.Other.Other
d__Bacteria.p__Firmicutes.c__Erysipelotrichia.o__Erysipelotrichales
d__Bacteria.p__Firmicutes.c__Erysipelotrichia
d__Bacteria.p__Firmicutes.c__Bacilli.o__Lactobacillales.f__Lactobacillaceae
d__Bacteria.p__Firmicutes.c__Bacilli.o__Lactobacillales
d__Bacteria.Other.Other.Other
d__Bacteria.p__Bacteroidetes.c__Bacteroidia.o__Bacteroidales
d__Bacteria.Other
d__Bacteria.p__Proteobacteria.c__Deltaproteobacteria.o__Desulfovibrionales
d__Bacteria.Other.Other.Other.Other.Other
d__Bacteria.p__Firmicutes.c__Bacilli.o__Lactobacillales.f__Lactobacillaceae.g__Lactobacillus
d__Bacteria.p__Bacteroidetes.c__Bacteroidia.o__Bacteroidales.f__Rikenellaceae
d__Bacteria.p__Bacteroidetes.c__Bacteroidia.o__Bacteroidales.f__Rikenellaceae.g__Alistipes
d__Bacteria.p__Proteobacteria
d__Bacteria.p__Proteobacteria.c__Deltaproteobacteria.o__Desulfovibrionales.Other
d__Bacteria.Other.Other.Other.Other
d__Bacteria.p__Proteobacteria.c__Deltaproteobacteria.o__Desulfovibrionales.Other.Other
d__Bacteria.p__Firmicutes.c__Bacilli
d__Bacteria.Other.Other
d__Bacteria.p__Proteobacteria.c__Deltaproteobacteria
D
LDA Score
WT Gut B cell
IgA -/- Gut B cell
d__Bacteria.p__Firmicutes
d__Bacteria.p__Firmicutes.Other.Other.Other.Other
d__Bacteria.p__Firmicutes.Other.Other
d__Bacteria.p__Firmicutes.Other.Other.Other
d__Bacteria.p__Firmicutes.Other
d__Bacteria.p__Firmicutes.c__Clostridia.o__Clostridiales.f__Lachnospiraceae.Other
d__Bacteria.p__Firmicutes.c__Erysipelotrichia.Other
d__Bacteria.p__Firmicutes.c__Erysipelotrichia.Other.Other.Other
d__Bacteria.p__Firmicutes.c__Erysipelotrichia.Other.Other
d__Bacteria.p__Firmicutes.c__Bacilli.o__Lactobacillales.f__Lactobacillaceae
d__Bacteria.p__Firmicutes.c__Bacilli.o__Lactobacillales
d__Bacteria.p__Proteobacteria.c__Gammaproteobacteria
d__Bacteria.p__Firmicutes.c__Clostridia.o__Clostridiales.f__Clostridiaceae_1
d__Bacteria.p__Firmicutes.c__Erysipelotrichia.o__Erysipelotrichales.f__Erysipelotrichaceae
d__Bacteria.p__Firmicutes.c__Bacilli.o__Lactobacillales.f__Lactobacillaceae.g__Lactobacillus
d__Bacteria.p__Bacteroidetes.Other.Other.Other.Other
d__Bacteria.p__Proteobacteria.c__Gammaproteobacteria.o__Pseudomonadales.f__Pseudomonadaceae.g__Pseudomonas
d__Bacteria.p__Bacteroidetes.Other
d__Bacteria.p__Proteobacteria.c__Gammaproteobacteria.o__Pseudomonadales
d__Bacteria.p__Bacteroidetes.Other.Other
d__Bacteria.p__Firmicutes.c__Clostridia.o__Clostridiales.f__Lachnospiraceae.g__Clostridium_XlVb
d__Bacteria.p__Firmicutes.c__Clostridia.o__Clostridiales.f__Clostridiaceae_1.g__Clostridium_sensu_stricto
d__Bacteria.p__Proteobacteria
d__Bacteria.p__Firmicutes.c__Erysipelotrichia.o__Erysipelotrichales.f__Erysipelotrichaceae.g__Clostridium_XVIII
d__Bacteria.p__Bacteroidetes.Other.Other.Other
d__Bacteria.p__Firmicutes.c__Bacilli
d__Bacteria.p__Proteobacteria.c__Gammaproteobacteria.o__Pseudomonadales.f__Pseudomonadaceae
d__Bacteria.p__Firmicutes.c__Erysipelotrichia.o__Erysipelotrichales
d__Bacteria.p__Firmicutes.c__Erysipelotrichia
d__Bacteria.p__Firmicutes.c__Clostridia.o__Clostridiales.f__Lachnospiraceae.g__Acetatifactor
d__Bacteria.p__Bacteroidetes
E
PCoA: Unweighted UniFrac
Axis.1 [29.3%]
Axis.2 [23.8%]
MousexSample
KB_LB
KO_cecal
KO_LB
KO_SB
WB_LB
WT_cecal
WT_LB
WT_SB
F
KB_LB (n=3)
KO_LB (n=3)
KO_SB (n=3)
KO_cecal (n=3)
MC (n=1)
PAO1 (n=2)
WB_LB (n=3)
WT_LB (n=3)
WT_SB (n=3)
WT_cecal (n=3)
G
Total % IgA-bound Bacteria in Distal SB
% Highly-bound IgA Bacteria in Distal SB
NCD
HFD

Supplementary Figure 8. Metabolic testing of HFD fed germ free mice transplanted with IgA-/- fecal matter, gut microbiota LEfSe analysis of IgA-deficient mice fed HFD and B cell adoptive transfer mice, and IgA bound bacteria in ileal contents. A) Fasting glucose (left)**,** glucose tolerance (also represented by AUC) (middle), and insulin tolerance (also represented by AUC) (right) of HFD fed germ free transplanted with fecal matter from HFD fed IgA$^{-/-}$ and IgA^{wt} mice. (n=3 for WT and 4 for IgA$^{-/-}$ transplanted group). **(B)** Bacterial 16S Ct values from feces of HFD-fed antibiotic treated mice (top) (n=5/group) and germ free mice (bottom) transplanted with fecal matter from HFD fed IgA$^{-/-}$ and IgA^{wt} mice (n=3 WT FT, 4 IgA$^{-/-}$ FT and 8 GF WT mice). **(C-D)** Significant taxa from LEfSe analysis of colon bacterial communities in **C)** IgA$^{-/-}$ vs. WT HFD mice and **D)** HFD-fed B-cell deficient (µMT$^{-/-}$) mice adoptively transferred with either IgA$^{-/-}$ or WT intestinal pan B cells (n=3/group; LDA score > 2, p-value < 0.05). **E)** Principal coordinate analysis (PCoA) of bacterial communities within IgA$^{-/-}$, WT or B cell adoptive transfer recipient mice (µMT$^{-/-}$ mice receiving WT or IgA$^{-/-}$ intestinal B cells indicated by WB or KB, respectively) (n=3/group). **F)** Alpha diversity (Chao1) plots of bacterial communities within IgA$^{-/-}$, WT or B cell adoptive transfer recipient mice (Green underline – IgA$^{-/-}$ vs. WT intestinal B cell adoptively transferred µMT$^{-/-}$ mice colon microbiota, Red - IgA$^{-/-}$ vs. WT colon microbiota, Blue - IgA$^{-/-}$ vs. WT distal small intestine microbiota, Yellow - IgA$^{-/-}$ vs. WT cecal microbiota) (n=3/group). **G)** Percentage of total (top) and high-affinity IgA-bound bacteria (bottom) within the ileal contents of HFD-fed mice compared to NCD controls (n=5 NCD, 4-5 HFD). Data are means ± SEM. * denotes p<0.05, ** denotes p<0.01 and *** denotes p<0.001.

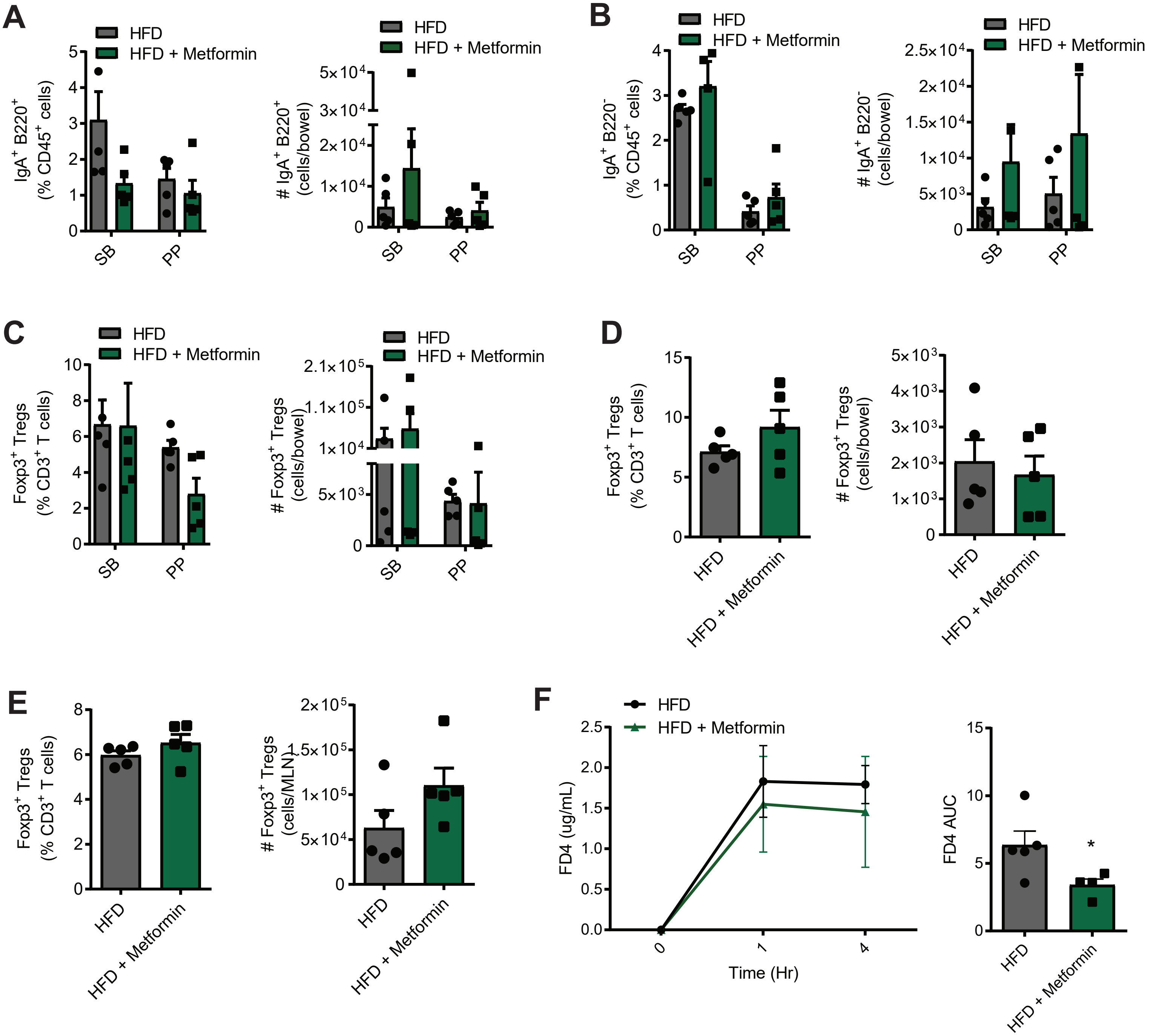

Supplementary Figure 9. IgA producing populations and Tregs in the small bowel and Peyer's patches, and intestinal permeability of HFD + metformin treated WT mice. Frequency (left) and absolute numbers (right) of IgA producing **A)** B cells ($B220^{+}$), **B)** plasma cells ($B220^{-}$) and **C)** regulatory T cells (Tregs) in the small bowel and Peyer's patches of HFD-fed mice treated with or without metformin (300mg kg^{-1} per day) in drinking water for 14 weeks (n=5/group). Frequency (left) and absolute number (right) of Tregs in the **D)** colon and **E)** MLN of metformin-treated HFD-fed mice (n=5/group). **F)** Intestinal permeability assay measuring concentration of FITC labelled-dextran by fluorescence (left) also represented by area under the curve (AUC) (right) in collected plasma at 1 and 4 hours post-gavage in HFD-fed WT mice treated with or without metformin (n=4-5/group). Data are means ± SEM. * denotes $p<0.05$.

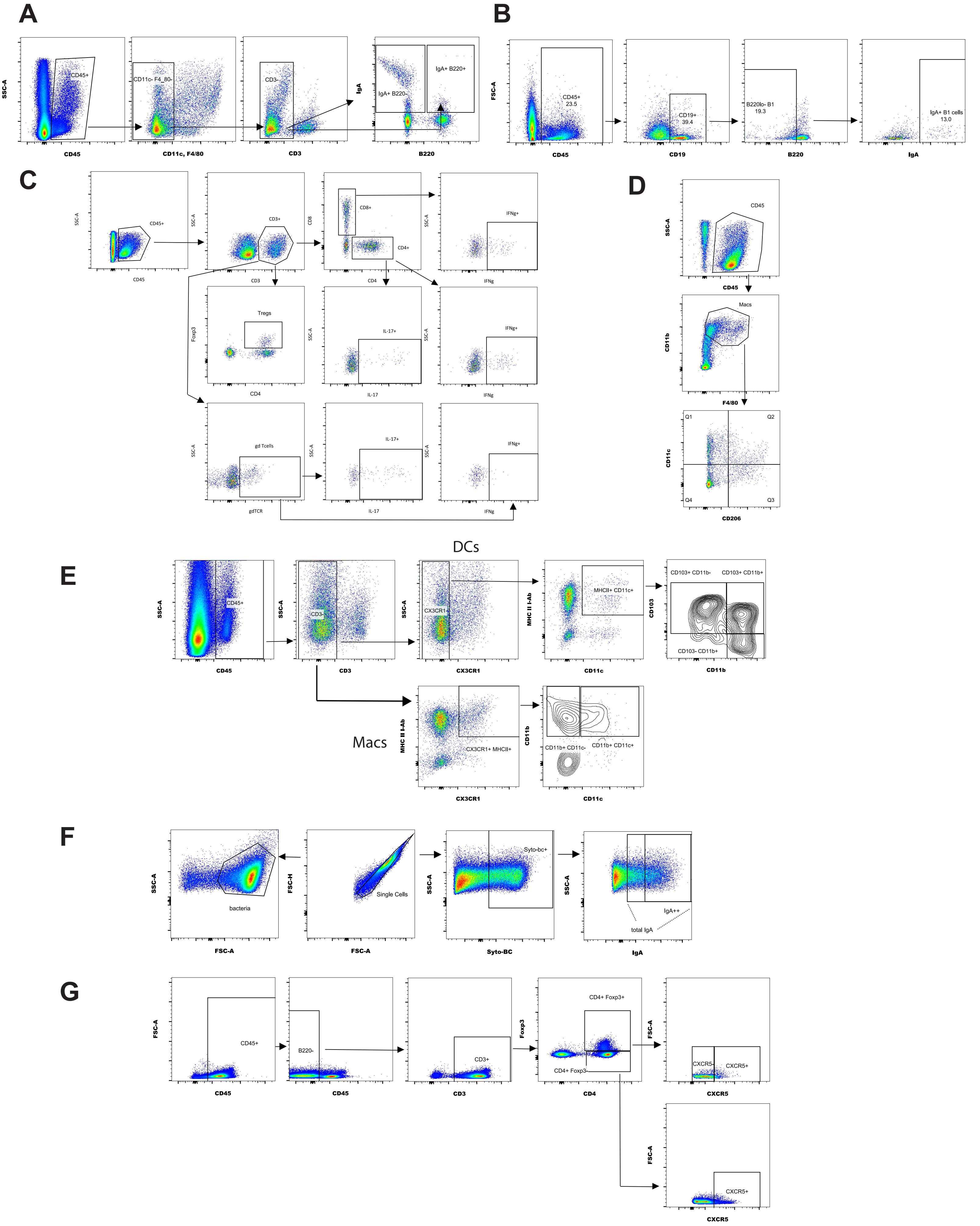

A
SSC-A
CD45+
CD45
CD11c- F4_80-
CD11c, F4/80
CD3-
CD3
IgA
IgA+ B220-
IgA+ B220+
B220
B
FSC-A
CD45+
23.5
CD45
CD19+
39.4
CD19
B220lo- B1
19.3
B220
IgA+ B1 cells
13.0
IgA
C
SSC-A
CD45+
CD45
CD3+
CD3
CD8
CD8+
CD4+
CD4
IFNg+
IFNg
Tregs
Foxp3
IL-17+
IL-17
gd Tcells
gdTCR
D
CD45
Macs
CD11b
F4/80
Q1
Q2
Q3
Q4
CD11c
CD206
DCs
E
CD45+
CD3-
CX3CR1-
CX3CR1
MHC II I-Ab
MHCII+ CD11c+
CD11c
CD103
CD103+ CD11b-
CD103+ CD11b+
CD103- CD11b+
CD11b
Macs
CX3CR1+ MHCII+
CD11b+ CD11c-
CD11b+ CD11c+
F
SSC-A
bacteria
FSC-A
FSC-H
Single Cells
Syto-bc+
Syto-BC
IgA++
total IgA
IgA
G
FSC-A
CD45+
CD45
B220-
CD3+
CD3
Foxp3
CD4+ Foxp3+
CD4+ Foxp3-
CD4
CXCR5-
CXCR5+
CXCR5

Supplementary Figure 10. Gating strategy used for flow cytometry analysis of immune cell populations. A) Gating strategy for IgA producing immune cell populations presented in Figure 1A-F and Figure 8A-D. **B)** Gating strategy for IgA producing B1 cells in Supplementary Figure 1D. **C)** Gating strategy for stimulated T cell populations with IFNγ and IL-17 cytokines, and unstimulated regulatory T cells presented in Figure 4A-H and 5D, Supplementary Figure 4, 5, 6C-G, 7D-G. **D)** Gating strategy for macrophage populations within the visceral adipose tissue presented in Figure 5C and Supplementary Figure 6A. **E)** Gating strategy of gut-associated dendritic cell and macrophage total population and their subsets presented in Figure 2 D-G and Supplementary Figure 2 D-G, I-L, N-Q. **F)** Gating strategy for bacterial flow cytometry for IgA-bound bacteria and high affinity bound bacteria from luminal stool presented in Figure 7 H-I and Supplementary Figure 8G. **G)** Gating strategy for follicular T helper (Tfh) and follicular T regulatory cells (Tfr) presented in Figure 7J.

Supplementary Table 1. Bariatric surgery human cohort characteristics at baseline and 1 month post-surgery (n=14). Data ± SEM.

		Baseline			1 Month Post-Surgery		
Age (Yrs)	Gender	Weight (kg)	BMI (kg/m2)	HOMA-IR score	Weight (kg)	BMI (kg/m2)	HOMA-IR score
52.6 ± 1.7	(3M:11F)	128.8 ± 5.6	46.8 ± 1.5	5.8 ± 0.8	113.2 ± 4.4	40.6 ± 1.5	2.4 ± 0.4

Supplementary Table 2. Oligonucleotide sequences for SYBR qPCR assays and 16S bacterial load qPCR

Oligonucleotide	Sequence (5' – 3')
Primer: *GAPDH* forward	TGTGTCCGTCGTGGATCTGA
Primer: *GAPDH* reverse	CCTGCTTCACCACCTTCTTGA
Primer: *Aldh1a1* forward	CTCCTCTCACGGCTCTTCA
Primer: *Aldh1a1* reverse	AATGTTTACCACGCCAGGAG
Primer: *Aldh1a2* forward	GACTTGTAGCAGCTGTCTTCACT
Primer: *Aldh1a2* reverse	TCACCCATTTCTCTCCCATTTCC
Primer: *Tgfb1* forward	GCAACATGTGGAACTCTACCAGA
Primer: *Tgfb1* reverse	GACGTCAAAAGACAGCCACTCA
Primer: *Il5* forward	ATGGAGATTCCCATGAGCAC
Primer: *Il5* reverse	AGCCCCTGAAAGATTTCTCC
Primer: *Tnfsf13* forward	TCACAATGGGTCAGGTGGTATC
Primer: *Tnfsf13* reverse	TGTAAATGAAAGACACCTGCACTGT
Primer: *Tnfsf13b* forward	TGCTATGGGTCATGTCATCCA
Primer: *Tnfsf13b reverse*	GGCAGTGTTTTGGGCATATTC
Primer: *Ccl2* forward	CCCAATGAGTAGGCTGGAGA
Primer: *Ccl2* reverse	TCTGGACCCATTCCTTCTTG
Primer: *Il10* forward	GGTTGCCAAGCCTTATCGGA
Primer: *Il10* reverse	ACCTGCTCCACTGCCTTGCT
Primer: *Il6* forward	GGTACATCCTCGACGGCATCT
Primer: *Il6* reverse	GTGCCTCTTTGCTGCTTTCAC
Primer: *Tnfa* forward	GCCTCTTCTCATTCCTGCTTG
Primer: *Tnfa* reverse	CTGATGAGAGGGAGGCCATT
Primer: *16s* forward	TCCTACGGGAGGCAGCAGT
Primer: *16s* reverse	GGACTACCAGGGTATCTAATCCTGTT
Probe: *16s*	(6-FAM)- CGTATTACCGCGGCTGCTGGCAC-(NFQ-MGB)
