# Supplementary material for: Gut-associated IgA+ immune cells regulate obesity-related insulin resistance
Source: Nat Commun. 2019 Aug 13;10:3650. doi: 10.1038/s41467-019-11370-y (PMC6692361; doi:10.1038/s41467-019-11370-y)
Supplement: Supplementary file 2 — Reporting Summary [file 41467_2019_11370_MOESM2_ESM.pdf]

nature research

Corresponding author(s): Daniel A. Winer

Last updated by author(s): 2019/06/27

Reporting Summary

Nature Research wishes to improve the reproducibility of the work that we publish. This form provides structure for consistency and transparency in reporting. For further information on Nature Research policies, see Authors & Referees and the Editorial Policy Checklist.

Statistics

For all statistical analyses, confirm that the following items are present in the figure legend, table legend, main text, or Methods section.

n/a	Confirmed	
☐	☒	The exact sample size (*n*) for each experimental group/condition, given as a discrete number and unit of measurement
☐	☒	A statement on whether measurements were taken from distinct samples or whether the same sample was measured repeatedly
☐	☒	The statistical test(s) used AND whether they are one- or two-sided<br>*Only common tests should be described solely by name; describe more complex techniques in the Methods section.*
☒	☐	A description of all covariates tested
☐	☒	A description of any assumptions or corrections, such as tests of normality and adjustment for multiple comparisons
☐	☒	A full description of the statistical parameters including central tendency (e.g. means) or other basic estimates (e.g. regression coefficient) AND variation (e.g. standard deviation) or associated estimates of uncertainty (e.g. confidence intervals)
☒	☐	For null hypothesis testing, the test statistic (e.g. *F*, *t*, *r*) with confidence intervals, effect sizes, degrees of freedom and *P* value noted<br>*Give P values as exact values whenever suitable.*
☒	☐	For Bayesian analysis, information on the choice of priors and Markov chain Monte Carlo settings
☒	☐	For hierarchical and complex designs, identification of the appropriate level for tests and full reporting of outcomes
☒	☐	Estimates of effect sizes (e.g. Cohen's *d*, Pearson's *r*), indicating how they were calculated

Our web collection on statistics for biologists contains articles on many of the points above.

Software and code

Policy information about availability of computer code

Data collection	BD FACS Diva and BD LSRFortessa X-20 (flow cytometry), Quant Studio Flex 6 Real-Time PCR System (qPCR), BioTek Synergy HT and Gen5 (plate reader and software), Oxymax-CLAMS and Oxymax Systems (metabolic cages), Zen Blue and Zeiss AxioImager (immunofluoresence), MiSeq Ilumina (16S rRNA sequencing), Leica DFC320 camera with Leica application suite (LAS) software (immunohistochemistry)
Data analysis	FlowJo V10 (flow cytometry), GraphPad Prism 7 (data analysis), ImageJ (imaging analysis)

For manuscripts utilizing custom algorithms or software that are central to the research but not yet described in published literature, software must be made available to editors/reviewers. We strongly encourage code deposition in a community repository (e.g. GitHub). See the Nature Research guidelines for submitting code & software for further information.

Data

Policy information about availability of data

All manuscripts must include a data availability statement. This statement should provide the following information, where applicable:

- Accession codes, unique identifiers, or web links for publicly available datasets
- A list of figures that have associated raw data
- A description of any restrictions on data availability

The data availability statement is provided in our manuscript. The data that support the findings of this study can be found in the Source Data file and is available from the corresponding author upon reasonable request. Sequencing data is deposited in public database with accession code provided.

Field-specific reporting

Please select the one below that is the best fit for your research. If you are not sure, read the appropriate sections before making your selection.

☒ Life sciences ☐ Behavioural & social sciences ☐ Ecological, evolutionary & environmental sciences

For a reference copy of the document with all sections, see nature.com/documents/nr-reporting-summary-flat.pdf

Life sciences study design

All studies must disclose on these points even when the disclosure is negative.

Sample size	Sample size was not predetermined for either mouse or human studies, but we performed experiments with group sizes based on existing published literature of similar experiments.
Data exclusions	Grubbs' Test was performed to assess for statistical outliers.
Replication	Experimental and biological replicates for each figure are indicated in the figure legends and described in the methods.
Randomization	Animals were randomly assigned to either HFD (research Diets, 60 kcal% fat) or NCD (Envigo, 16 kcal% fat) starting at 6 weeks of age.
Blinding	Investigators were not blinded for glucose and metabolic testing or flow cytometry and qPCR analyses, but were blinded for analysis of histological specimens.

Reporting for specific materials, systems and methods

We require information from authors about some types of materials, experimental systems and methods used in many studies. Here, indicate whether each material, system or method listed is relevant to your study. If you are not sure if a list item applies to your research, read the appropriate section before selecting a response.

Materials & experimental systems

n/a	Involved in the study	
☐	☒	Antibodies
☒	☐	Eukaryotic cell lines
☒	☐	Palaeontology
☐	☒	Animals and other organisms
☐	☒	Human research participants
☒	☐	Clinical data

Methods

n/a	Involved in the study	
☒	☐	ChIP-seq
☐	☒	Flow cytometry
☒	☐	MRI-based neuroimaging

Antibodies

Antibodies used	The following antibodies (with dilution factor and catalogue number) were utilized for our study (all purchased from Biolegend unless indicated):<br><br>CD45.2 (1:200; cat#109822), CD3 (1:100; Cat#100209), CD4 (1:100; cat#100422), CD8 (1:100; cat#100710), γδTcR (1:50; cat# 118118), Foxp3 (1:50; cat#320012), IL-17 (1:100; cat#506903), IFNγ (1:50; cat#505830), CD11b (1:125; Cat#101208), F4/80 (1:100; Cat#123120), CD11c (1:125; Cat#117348), CD19 (1:100; Cat#115512), B220 (1:100; Cat#103258), IgD (1:200; Cat#405714), IgM (1:50; Cat#406531), IgG (1:100; Cat#406001), IgA (1:50; Thermo Fisher Scientific cat#12-4204-82), CD206 (1:50; Cat#141710), CD80 (1:100; cat#104708), CD86 (1:100; Cat#105011), CX3CR1 (1:50; Cat#149027), CD103 (1:50; cat#121416), CXCR5 (1:50; Cat#145512), and I-Ab MHC Class II (1:125; Cat#116420)<br><br>Immunofluoresence: MUC2 (anti-MUC2 antibody (Novus Biologicals), anti-rabbit Alexa488 secondary antibody (Invitrogen)<br><br>Immunohistochemistry: IgA primary antibody (NSJ Bioreagents R20169), secondary antibody biotinylated Anti-Rabbit Ig (Vector Labs Cat. No. BA-1000)
Validation	All antibodies were carefully selected for its desired applications and are commercially available. The antibodies were used according to manufacturer's instructions and validation details are available on suppliers' website.

Animals and other organisms

Policy information about studies involving animals; ARRIVE guidelines recommended for reporting animal research

Laboratory animals	WT and IgA-/- mice were generated via in-house littermate breeding of C57BL/6J (Jax 664) mice purchased from Jackson

Laboratory animals	Laboratory and C57BL/6J IgA-/- mice obtained from our collaborator, Margaret E. Connor (Baylor College of Medicine, USA). C57BL/6 B cell-deficient µMT-/- or Bnull mice (Jax 2288) were purchased from Jackson Laboratory. IgA-/- mice and littermates were weaned and separated according to genotype to control for IgA transmission. We obtained germ-free mice from McMaster University's germ-free facility, which were housed and maintained accordingly in the University of Toronto germ-free facility. Mice were maintained in a pathogen-free, temperature controlled, and 12h light and dark cycle environment at the Toronto Medical Discovery Tower animal research facility. We utilized diet-induced obesity (DIO) mouse models to examine metabolic disease in the various groups of mice assessed. All mice used in comparative studies were male, age-matched, littermates, and randomly assigned to either HFD (research Diets, 60 kcal% fat) or NCD (Envigo, 16 kcal% fat) starting at 6 weeks of age.
Wild animals	N/A
Field-collected samples	N/A
Ethics oversight	All animal studies were conducted under the guidelines of and approved under the Animal Use Protocol (2570.20) by the Animal Care Committee at the University Health Network.

Note that full information on the approval of the study protocol must also be provided in the manuscript.

Human research participants

Policy information about studies involving human research participants

Population characteristics	The study included 14 bariatric surgery patients, 3 males and 11 females. Average population characteristics including age, weight, HOMA-IR score, BMI of patients pre and 1-month post surgery can be found in Supplementary Table 1.
Recruitment	Patients were approached and consented for research after the surgeons confirmed that the patients were suitable for bariatric surgery according to the criteria stated by the National Institutes of Health. Patients were recruited at the University Health Network's Bariatric Clinic.
Ethics oversight	Human studies were performed with study approval by the Research Ethics Board for Human Subjects (#15-8784) at UHN with informed consent from all human subjects.

Note that full information on the approval of the study protocol must also be provided in the manuscript.

Flow Cytometry

Plots

Confirm that:

☒ The axis labels state the marker and fluorochrome used (e.g. CD4-FITC).

☒ The axis scales are clearly visible. Include numbers along axes only for bottom left plot of group (a 'group' is an analysis of identical markers).

☒ All plots are contour plots with outliers or pseudocolor plots.

☒ A numerical value for number of cells or percentage (with statistics) is provided.

Methodology

Sample preparation	Please refer to following sections in Methods: Isolation of Bowel Associated Immune Cells & Epithelial Cells; Isolation of Stromal Vascular Cells from Visceral Adipose Tissue; Flow Cytometry.<br>Briefly, single-cell suspensions were stained for 30 minutes on ice with fluorophore-conjugated commercial antibodies following viability cell stains with DAPI or nuclear stains with Zombie NIR or UV (Biolegend). Intracellular staining was performed using a Foxp3 staining buffer kit (eBioscience).
Instrument	Data was collected on a LSRFortessa X-20 flow cytometer (BD Biosciences)
Software	Data Collection: BD FACSDiva; Data Analysis: FlowJo V10 (Tree Star)
Cell population abundance	Pan B cells (includes plasma cells) from the spleens, Peyer's patches, MLN, and small and large intestinal LP of 14-16 weeks HFD-fed WT and IgA-/- mice were purified using negative selection (>90% purity, EasySep; StemCell Technologies). For immune cell population profiling, tissues were prepared and single cell suspensions were isolated as described in Methods followed by staining with markers including CD45 to assess for immune cell abundance. All cell frequencies are presented as % of CD45+ cells or subsequent gated populations in figures.
Gating strategy	Detailed gating strategies for each flow cytometry analysis is provided in Supplementary Figure 10

☒ Tick this box to confirm that a figure exemplifying the gating strategy is provided in the Supplementary Information.
